# Trends in insufficient physical activity among adults in China 2010–18: a population-based study

**DOI:** 10.1186/s12966-023-01470-w

**Published:** 2023-07-17

**Authors:** Mei Zhang, Yanan Ma, Xili Xie, Ming Sun, Zhengjing Huang, Zhenping Zhao, Xiao Zhang, Chun Li, Xingxing Gao, Jing Wu, Limin Wang, Maigeng Zhou, Deliang Wen

**Affiliations:** 1 National Center for Chronic and Non-Communicable Disease Control and Prevention, Chinese Center for Disease Control and Prevention, No.27 Nanwei Road, Xicheng District, Beijing, 100050 People’s Republic of China; 2 Department of Biostatistics and Epidemiology, School of Public Health, China Medical University, No.77 Puhe Road, Shenyang, Liaoning Province 110122 People’s Republic of China; 3 Health Sciences Institute, China Medical University, No.77 Puhe Road, Shenyang, Liaoning Province 110122 People’s Republic of China; 4 Liaoning Key Laboratory of Obesity and Glucose/Lipid Associated Metabolic Diseases, China Medical University, No. 77 Puhe Road, Shenyang, Liaoning Province 110122 People’s Republic of China

**Keywords:** Domain-specific physical activity, Insufficient physical activity, Moderate- to vigorous-intensity physical activity (MVPA), Temporal changes

## Abstract

**Background:**

The global prevalence of insufficient physical activity (PA) was reported to be 27.5% in 2016, and there were stable levels of insufficient PA worldwide between 2001 and 2016. The global target of a 10% reduction in insufficient PA by 2025 will not be met if the trends remain. The relevant data for trends in China were still scarce. This study aimed to determine nationwide temporal trends in insufficient PA among adults in China from 2010 to 2018.

**Methods:**

645 903 adults aged 18 years or older were randomly selected from four nationally representative cross-sectional surveys of the China Chronic Disease and Risk Factor Surveillance conducted in 2010, 2013, 2015, and 2018. PA was measured using the Global Physical Activity Questionnaire. Temporal changes in insufficient PA prevalence and participation of domain-specific moderate- to vigorous-intensity PA (MVPA) were analyzed using logistic regression.

**Results:**

From 2010 to 2018, the age-adjusted prevalence of insufficient PA in China increased from 17.9% (95% confidence interval 16.3% to 19.5%) in 2010 to 22.3% (20.9% to 23.8%) in 2018 (*P* for trend < 0.001). By age group, with a significant increase in insufficient PA in adults aged 18–34 years (*P* for trend < 0.001), which rose more rapidly than in adults aged ≥ 35 years (*P* for interaction < 0.001). Insufficient PA has increased significantly among adults engaged in agriculture-related work, non-manual work, and other manual work (all *P* for trend < 0.05). And among the occupational groups, those engaged in agriculture-related work had the fastest increase (*P* for interaction = 0.01). The percentage of adults participating in work-related MVPA decreased from 79.6% (77.8% to 81.5%) to 66.8% (64.9% to 68.7%) along with a decrease in time spent on work-related MVPA, while percentages of adults participating in recreation-related MVPA increased from 14.2% (12.5% to 15.9%) to 17.2% (16.0% to 18.4%) (all *P* for trend < 0.05).

**Conclusions:**

Among Chinese adults, an increasing trend was found in insufficient PA from 2010 to 2018, with more than one-fifth of adults failing to achieve the recommendation of adequate PA. More targeted PA promotion strategies should be developed to improve population health.

**Supplementary Information:**

The online version contains supplementary material available at 10.1186/s12966-023-01470-w.

## Introduction

Insufficient physical activity (PA), defined as less than 150 min of moderate-intensity aerobic PA or 75 min of vigorous-intensity aerobic PA per week, or equivalent [1, 2], is an important risk factor for non-communicable diseases (NCDs), including hypertension, type 2 diabetes, breast cancer, colorectal cancer, and chronic pain [3–9]. Insufficient PA also has been estimated as the fourth leading risk factor for global mortality [10] and is estimated to cost global healthcare systems $50 billion annually [11]. It has been a prominent and increasingly prevalent global public health problem [11, 12]. Since the end of the twentieth century, PA related recommendations highlighted moderate- to vigorous-intensity PA (MVPA), including walking, cycling, running, and gardening, mainly for adults [13]. MVPA can effectively weaken the link between sedentary time and the risk of cardiovascular disease mortality [14]. MVPA also improves mental health, such as depression and anxiety [15].

So far, many studies have explored the prevalence of insufficient PA and its trend over time, especially in developed countries. A global study of insufficient PA pooled data from 358 surveys across 168 countries and estimated global and regional trends in insufficient PA from 2001 to 2016. The results showed that in 2016, the prevalence of age-standardized insufficient PA was 27.5% globally, higher for women than men [16]. Another worldwide study found that insufficient PA is higher in high-income countries than in low-income [12]. Further, insufficient PA exists in several domains in life: at work, transport, or recreation, and different domains-specific PA may affect health differently [17]. Tessa et al. reported that work-related PA was the highest contributor, and recreation-related PA was the lowest contributor in PA of 81 countries, including China [18]. Otherwise, a previous study found that the odds of engaging in recreation-related PA among the elderly in China were higher than that of the younger [19]. Higher levels of all domain-specific PA were associated with a lower risk of all-cause mortality [17]. Recreation-related PA has a stronger relationship with mental health than work-related PA and transport-related PA [20]. It is essential to study domain-specific PA trends and propose appropriate policies. The Outline of the “Healthy China 2030” Plan, issued on October 25, 2016, has initiated a nationwide fitness campaign, stipulating that more than 40% of urban and rural residents should regularly participate in the exercise by 2030 [21, 22]. However, a long period is required to achieve the goal. To the best of our knowledge, no studies or reports provided temporal trends and detailed demographics, the location-specific prevalence of insufficient PA, and the PA patterns in China's total or subpopulation.

To further understand the changes in the prevalence of insufficient PA in the Chinese mainland in recent years, supplement the gaps in relevant domestic research fields, and provide a basis for future policy formulation and further research, our study used data from the China Chronic Disease and Risk Factor Surveillance (CCDRFS). It aimed to examine trends in insufficient PA among Chinese adults aged 18 years or older according to age, gender, geographical location, ethnicity, education, occupation, income, body mass index (BMI), and different domain-specific PA from 2010 to 2018.

## Methods

### Study design and population

**Fig. 1:**
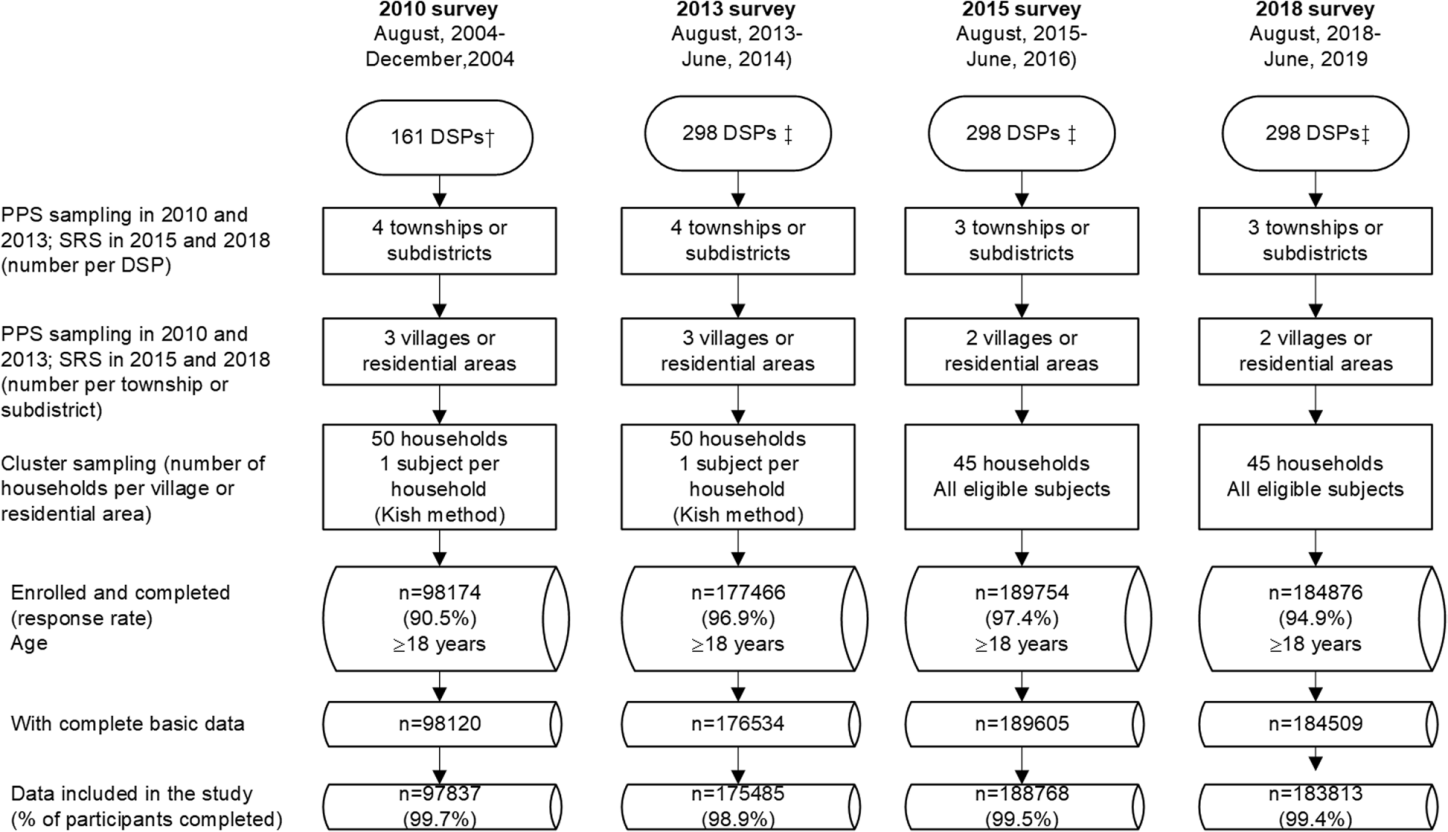
Flow diagram of study design and sampling procedure of China Chronic Disease and Risk Factors Surveillance 2010-18. DSPs: Disease Surveillance Points; PPS: probability to size sampling; SRS: systematic random sampling. †All DSPs were selected. ‡half of all DSPs (605) were selected using stratified sampling

The China Chronic Disease and Risk Factor Surveillance (CCDRFS), established by the Chinese Center for Disease Control and Prevention (China CDC) and incorporated into the National Disease Surveillance Points (DSPs) system, is a series of periodical cross-sectional nationwide surveys that collected data on PA and other health-related risk factors (e.g., smoking, harmful drinking, and unbalanced diet) [23]. DSPs system comprised 161 districts/counties in all 31 provinces (autonomous and municipalities) from 2004 to 2012 and expanded to 605 districts/counties since 2013 [24, 25]. Within the DSPs scheme, the CCDRFS has also been designed to represent mainland China's entire population. In 2010, all 161 DSPs conducted the CCDRFS survey. Since 2013, with the expansion of the DSPs system, the CCDRFS was further expanded to 298 DSPs and represented the national and provincial levels (Appendix 1). Appendix Fig. 1 shows the maps of surveillance districts/counties before and after expansion in 2013. Within the DSPs, the participants were selected using a multi-stage stratified clustering random sampling method. First, the townships or subdistricts were chosen using the proportional to population size (PPS) sampling (2010 and 2013) or the systematic random sampling (SRS, 2015 and 2018) [26]. Second, the villages or residential areas were selected using the PPS (2010 and 2013) method or SRS (2015 and 2018) method in each chosen township/subdistrict. Third, each village or residential area was divided into several residential quarters, with 50–60 households. Finally, 50 families from one residential quarter were selected to be the target households in 2010 and 2013, and 45 families from one residential quarter were selected to be the target households in 2015 and 2018 (Fig. 1). For the CCDRFS 2010 and CCDRFS 2013, the Kish method was used to choose one participant within every household [27, 28]. Since 2015, all eligible participants were invited to participate in the field survey. The participants who met the following inclusion criteria were included in the CCDRFS: 1) aged 18 years or older; 2) having lived in the address for more than six months in the past 12 months; 3) not pregnant; 4) with no serious health condition or illness which could be the inability to complete the interviews or potentially interfere with PA, including intellectual disability or language disorder. Figure 1 shows the details of the sampling frame of four CCDRFS surveys. The Ethical Committee of the Chinese Center for Disease Control and Prevention (China CDC) approved the 2015 survey (No.201519-B). All other CCDRFS surveys (No.201010, No.201307, No.201819) were approved by the Ethical Committee of the National Center for Chronic and Noncommunicable Disease Control and Prevention (NCNCD), except the 2015 survey. Every participant provided written informed consent. The Ethical Committee of the NCNCD approved this study to analyze all CCDRFS data (202019).

Of the 681 236 individuals initially invited, 650 270 completed the surveys (95.5%), including 98 174 in 2010, 177 466 in 2013, 189 754 in 2015, and 184 876 in 2018. The corresponding survey response rates from 2010 to 2018 were 90.5%, 96.9%, 97.4%, and 94.9%, respectively. After excluding 1564 participants with missing basic information data (e.g., age, gender, educational attainment, etc.) and 2803 participants with missing or unreasonable values for PA, 645 903 adults were included in this study.

### Physical activity measures

Trained interviewers from the local Centers for Disease Control and Prevention conducted the face-to-face questionnaire interview, physical measurement, and biochemical sample collection. The householder or an adult well acquainted with the household was interviewed using a household questionnaire. The economic and environmental information of the household, as well as the basic information of all family members (e.g., birth date, gender), was collected. The participants meeting the inclusion criteria were interviewed by investigators using an individual questionnaire (paper-and-pencil based in 2010 and 2013, tablet-assisted in 2015 and 2018) to obtain information on demographic characteristics, lifestyle factors, and history of chronic diseases (Appendix 2). All data collected by paper–pencil interviews were entered in customized entry software promptly by interviewees and were delivered to the NCNCD by the internet. Once outliers or missing data are found during the entry process, the interviewees were required to correct or supplement them in time. No significant difference in the number of outliers or missing values was found between the data collected by the paper-and-pencil interviewing and tablet-assisted interviewing.

The Global Physical Activity Questionnaire (GPAQ) released by World Health Organization (WHO) was used to collect data on PA in the CCDRFS since 2010 (Appendix 4&5)[29, 30]. The GPAQ is a global tool for measuring PA and has been validated among adults in more than 20 countries. Short-term (that is, every one to two weeks) and long-term (that is, two to three months apart) test–retest reliability was good to very good [31]. Therefore, we used the data from 2010 to 2018 in the present study to ensure the comparability of the results. The GPAQ measures PA in duration, frequency, intensity, and context (work, transport, and leisure time) [32]. According to the GPAQ, PA is categorized into three modules: work-related (including household chores), transport-related, and recreation-related. Work-related and recreation-related PA were further divided into two subtypes with different intensities: vigorous and moderate. Transport-related PA was moderate-intensity. Participants were also asked to provide PA information, including the days engaged in a subtype of PA (e.g., vigorous work-related, moderate work-related, transport-related, vigorous recreation-related, and moderated recreation-related) in a typical week and time spent on a typical day. In 2010, the WHO recommended that all adults should do at least 150 min of moderate-intensity aerobic PA, at least 75 min of vigorous-intensity aerobic PA, or an equivalent combination of moderate- and vigorous-intensity activity throughout the week for substantial health benefits[33]. In 2013, insufficient PA was included in 25 indicators for NCDs comprehensive global monitoring framework and defined as less than 150 min of moderate-intensity activity per week, or equivalent[2]. The WHO released updated global guidelines on PA and sedentary behavior in 2020 [10]. In general, the minimum recommended amount of sufficient PA remains 150 min of moderate-intensity activity per week or equivalent. In this study, the participants who did not meet the minimum recommended amount of PA by WHO (< 150 min/week) were considered individuals with insufficient PA. When calculating the total time of MVPA, the time of vigorous PA should be multiplied by 2. We also reported the trends in the percentages of adults with MVPA more than 150 min/week and less than 300 min/week. The adults participating in domain-specific or intensity-specific PA were defined as having a ‘Yes’ response to the participation of PA subtypes. Age was classified as 18–34 years, 35–49 years, 50–64 years, and 65 years and above. According to the Compilation Rules of Statistical Zoning Code and Urban–Rural Division Code formulated by the National Bureau of Statistics, we used the urban and rural division codes to confirm whether the region where the participants live is urban or rural areas. Urban and rural classification codes beginning with a 1 indicate an urban community and beginning with a 2 indicate a rural village. Educational attainment was classified as secondary school or less, high school, and college or above. The occupations of the participants were classified into five groups: agriculture-related work, other manual work, non-manual work, unemployed (including students), and retired. BMI was categorized as underweight (BMI < 18.5 kg/m^2^), normal weight (18.5 kg/m^2^ ≤ BMI < 25 kg/m^2^), overweight (25 kg/m^2^ ≤ BMI < 30 kg/m^2^), and obese (BMI ≥ 30 kg/m^2^) according to WHO classification [34].

### Statistical analyses

We accounted for the complex sample design using Taylor's series method with finite population correction for primary sampling units while estimating the sampling error [35]. All estimates, including the prevalence and means, were age-adjusted to the 2010 China standard population. The trends in prevalence or percentages over the four survey cycles by gender, age, geographical location, education, ethnicity, occupation, income, and BMI group were tested using logistic regression models; the survey cycle was treated as a continuous (ordered categorical) and independent variable. The homogeneity of the prevalence of insufficient PA across strata in each survey cycle was tested by using logistic regression models including survey cycle × strata interaction terms, with strata-specific survey cycle effect models also fitted. To test linear trends of mean minutes per week of MVPA over time, we performed linear regression models with the survey year included as an independent variable. Statistical significance was determined as a two-sided *P* < 0.05. All statistical analyses were performed using SAS software version 9.4 (SAS Institute Inc, Cary, North Carolina, U.S.).

## Results

Table 1 shows the essential characteristics of the samples for each survey. Between 2010 and 2018, the number of participants included in each CCDRFS survey increased from 97 837 to 183 813. From 2010 to 2018, adults living in urban areas (51.7%) and those with college or above education (17.7%) showed a higher proportion in 2018 compared to those in 2010, whereas the inverse result was showed for adults with secondary school or less education (71.8% to 64.7%). From 2010 to 2018, a lower proportion of adults performed agriculture-related work (44.0% to 33.8%). Nevertheless, adults performed non-manual work (38.1% to 47.1%), and the unemployed adults/students (6.9% to 8.9%) were observed as a higher proportion. The proportion of adults with BMI ≥ 25 kg/m^2^ increased steadily.

**Table 1:** Characteristics of participants in each survey

Characteristics	2010 (*n* = 97 837)	2013 (*n* = 175 485)	2015 (*n* = 188 768)	2018 (*n* = 183 813)
Gender				
Men	44 734(50.5)	75 063(50.5)	88 590(50.5)	81 533(50.3)
Women	53 103(49.5)	100 422(49.5)	100 178(49.5)	102 280(49.7)
Age (years)				
18–34	21 805(34.6)	23 994(34.6)	28 399(34.7)	18 608(34.8)
35–49	35 395(33.1)	56 348(33.1)	53 629(33.2)	43 032(33.1)
50–64	28 496(20.9)	64 298(20.9)	69 576(20.8)	74 856(20.8)
≥ 65	12 141(11.4)	30 845(11.4)	37 164(11.3)	47 317(11.3)
Geographical location				
Urban	44 843(46.5)	80 992(46.0)	76 842(51.8)	74 896(51.7)
Rural	52 994(53.5)	94 493(54.0)	111 926(48.2)	108 917(48.3)
Ethnicity				
Han	832 242(91.2)	154 958(91.3)	165 189(91.9)	160 859(90.7)
Others	14 595(8.8)	20 527(8.7)	23 579(8.1)	22 954(9.3)
Education				
Secondary school or less	73 802(71.8)	139 378(72.2)	150 072(68.1)	146 985(64.7)
High school	15 943(18.0)	24 467(17.1)	24 472(17.1)	23 915(17.6)
College or above	8 092(10.2)	11 640(10.7)	14 224(14.8)	12 913(17.7)
Occupation				
Agriculture-related work	46 290(44.0)	82 221(42.4)	85 271(35.5)	80 546(33.8)
Other manual work	3 767(5.7)	7 984(6.3)	7 232(5.8)	5 783(4.9)
Non-manual work	34 965(38.1)	64 876(40.7)	69 972(45.5)	67 082(47.1)
Unemployed/students	4 923(6.9)	5 913(5.9)	9 187(7.9)	10 039(8.9)
Retired	7 892(5.4)	14 491(4.6)	17 106(5.3)	20 363(5.3)
Income per capita (¥)				
Q1	18 860(18.3)	33 505(18.3)	39 289(15.8)	34 096(15.2)
Q2	18 070(17.0)	27 549(16.8)	35 841(17.4)	31 501(15.8)
Q3	19 512(19.1)	39 466(23.3)	37 038(20.2)	36 555(20.7)
Q4	19 058(20.5)	33 549(17.5)	38 809(25.6)	38 497(24.9)
Refused/do not know	22 337(25.1)	41 416(24.1)	37 791(21.0)	43 164(23.4)
BMI category (kg/m^2^)				
< 18.5	3 854(4.7)	6 133(4.6)	6 730(4.5)	5 306(4.2)
18.5–24.9	59 610(62.3)	99 388(59.1)	105 154(58.7)	97 893(55.3)
25.0–29.9	26 668(27.8)	56 792(30.0)	57 964(30.2)	62 466(32.6)
≥ 30.0	5 510(5.2)	11 593(6.3)	11 409(6.6)	12 831(7.9)

Values are n (%) unless otherwise indicated. All percentages were weighted to represent the whole population aged 18 years or older. Values of polytomous variables may not sum to 100% due to rounding. For BMI subgroup analysis, 14 602 participants (195 in 2010, 1 579 in 2013, 7 511 in 2015, and 5 317 in 2018) were excluded due to missing or abnormal BMI values

### Insufficient PA prevalence

The overall prevalence of age-adjusted insufficient PA among Chinese adults aged 18 years or older increased from 17.9% (95% confidence interval 16.3 to 19.5) in 2010 to 22.3% (20.9 to 23.8) in 2018, with a slight decrease in 2013 (*P* for trend < 0.001). By gender, the prevalence of MVPA < 150 min/w increased from 20.1% (18.5 to 21.8) to 24.4% (23.0 to 25.9) among men, while an increase from 15.7% (13.9 to 17.4) to 20.2 (18.5 to 21.9) was observed among women (all *P* for trend < 0.001). By age group, with a significant increase of 6.1% in insufficient PA in younger adults aged 18–34 years (*P* for change < 0.001), which rose more rapidly than in adults aged ≥ 35 years (*P* for interaction < 0.001), but no statistically significant change was observed in the elderly (*P* for change = 0.13). The prevalence of insufficient PA in rural adults rose from 17.1% to 22.6%, while that rose from 18.8% to 22.0% in urban adults (all *P* for trend < 0.001). From 2010 to 2018, the prevalence of insufficient PA increased in both Han (from 18.1 to 22.5, *P* for trend < 0.001) and other ethnic groups (from 15.9 to 20.3, *P* for trend < 0.001). From 2010 to 2018, insufficient PA increased significantly among adults with lower educational qualifications (high school or less), those engaged in agriculture-related work, non-manual work, and other manual work (all *P* for trend < 0.05). In addition, among the occupational groups, those engaged in agriculture-related work had the fastest increase in the prevalence of insufficient PA (*P* for interaction = 0.01). Our results suggest that the prevalence of insufficient PA is on the rise across all income groups (all *P* for trend < 0.05). Since 2010, the prevalence of insufficient PA has increased among all BMI groups, especially among adults with a BMI < 18.5 kg/m^2^ (all *P* for trend < 0.05) (Table 2).

**Table 2:** Trends in insufficient PA (MVPA < 150 min/w) among adults in China, 2010–18

Characteristics	2010	2013	2015	2018	*P* for trend	*P* for interaction	Change in prevalence, 2018 vs. 2010	*P* for change
Total	17.9(16.3 to 19.5)	16.2(15.1 to 17.4)	21.2(20.0 to 22.4)	22.3(20.9 to 23.8)	< 0.001		4.4(2.4 to 6.4)	< 0.001
Gender								
Men	20.1(18.5 to 21.8)	18.1(16.9 to 19.4)	23.1(21.8 to 24.4)	24.4(23.0 to 25.9)	< 0.001	0.86	4.3(2.3 to 6.3)	< 0.001
Women	15.7(13.9 to 17.4)	14.3(13.0 to 15.6)	19.3(18.0 to 20.6)	20.2(18.5 to 21.9)	< 0.001		4.5(2.3 to 6.8)	< 0.001
Age group (years)								
18–34	20.0(18.1 to 21.8)	18.7(17.0 to 20.5)	25.2(23.7 to 26.8)	26.1(24.2 to 27.9)	< 0.001	< 0.001	6.1(3.7 to 8.6)	< 0.001
35–49	15.0(13.4 to 16.6)	13.5(12.4 to 14.6)	18.2(17.0 to 19.5)	19.9(18.2 to 21.6)	< 0.001		4.9(2.7 to 7.2)	< 0.001
50–64	14.0(12.2 to 15.7)	12.2(11.1 to 13.3)	16.1(14.8 to 17.3)	18.2(16.8 to 19.7)	< 0.001		4.2(2.1 to 6.4)	< 0.001
≥ 65	27.6(25.1 to 30.1)	23.9(22.3 to 25.6)	27.1(25.1 to 29.1)	25.5(23.9 to 27.1)	0.37		2.1(-4.8 to 0.6)	0.13
Geographical location								
Urban	18.8(17.0 to 20.6)	17.5(16.0 to 19.0)	21.4(20.3 to 22.5)	22.0(20.3 to 23.8)	< 0.001	0.15	3.2(0.8 to 5.6)	0.008
Rural	17.1(15.2 to 19.1)	15.1(13.7 to 16.6)	21.0(19.1 to 22.9)	22.6(20.7 to 24.6)	< 0.001		5.5(2.8 to 8.1)	< 0.001
Ethnicity								
Han	18.1(16.5 to 19.7)	16.2(15.2 to 17.3)	21.3(20.0 to 22.5)	22.5(21.0 to 24.1)	< 0.001	0.63	4.4(2.3 to 6.5)	< 0.001
Others	15.9(11.3 to 20.5)	16.1(11.2 to 20.9)	20.7(17.5 to 23.9)	20.3(16.9 to 23.8)	< 0.001		2.2(0.4 to 8.4)	0.029
Education								
Secondary school or less	16.8(15.1 to 18.6)	14.9(13.7 to 16.0)	20.6(19.2 to 22.0)	21.2(19.7 to 22.6)	< 0.001	0.25	4.3(2.2 to 6.4)	< 0.001
High school	19.2(17.4 to 21.0)	17.9(16.0 to 19.7)	21.7(20.2 to 23.2)	23.3(20.9 to 25.8)	0.001		4.2(1.2 to 7.1)	0.006
College or above	23.3(20.6 to 26.1)	22.9(20.4 to 25.4)	23.6(21.8 to 25.4)	25.5(22.9 to 28.2)	0.13		2.2(-1.2 to 5.7)	0.20
Occupation								
Agriculture-related work	13.6(11.7 to 15.5)	12.5(11.0 to 14.0)	18.1(16.0 to 20.1)	19.2(17.3 to 21.1)	< 0.001	0.01	5.6(3.1 to 8.1)	< 0.001
Other manual work	20.2(17.3 to 23.2)	18.9(16.5 to 21.4)	22.0(19.0 to 25.0)	24.0(21.4 to 26.6)	0.02		3.8(0.2 to 7.4)	0.041
Non-manual work	21.6(19.6 to 23.6)	18.9(17.5 to 20.3)	23.2(22.0 to 24.4)	24.3(22.5 to 26.0)	0.002		2.7(0.2 to 5.2)	0.037
Unemployed/students	25.8(21.9 to 29.7)	23.5(20.6 to 26.4)	27.6(24.9 to 30.3)	26.7(23.6 to 29.7)	0.46		0.9(- 3.7 to 5.5)	0.70
Retired	14.7(12.5 to 16.9)	13.7(11.7 to 15.8)	15.3(14.0 to 16.6)	16.0(14.6 to 17.4)	0.23		1.3(-1.2 to 3.8)	0.31
Income per capita (¥)								
Q1	17.1(14.2 to 20.1)	14.8(13.3 to 16.3)	21.7(19.7 to 23.8)	23.1(21.0 to 25.3)	< 0.001	0.10	6.0(3.0 to 9.0)	< 0.001
Q2	16.5(14.3 to 18.6)	14.0(12.7 to 15.3)	20.9(19.1 to 22.6)	22.1(19.5,24.6)	< 0.001		5.6(2.3 to 8.9)	0.001
Q3	16.5(14.7 to 18.3)	16.0(14.6 to 17.3)	20.3(18.7 to 21.8)	19.8(18.3,21.2)	0.001		3.3(1.2 to 5.3)	< 0.001
Q4	18.6(16.4 to 20.7)	17.0(15.5 to 18.5)	20.1(18.8 to 21.5)	22.6(20.8,24.4)	0.006		4.0(1.3 to 6.8)	0.004
Refused/do not know	21.5(18.9 to 24.1)	18.6(16.0 to 21.2)	23.4(21.6 to 25.3)	23.9(20.8,27.0)	< 0.001		2.4(1.6 to 6.5)	< 0.001
BMI category (kg/m^2^)								
< 18.5	21.2(18.9 to 23.6)	21.6(18.8 to 24.4)	26.2(23.8 to 28.7)	27.1(23.8 to 30.4)	< 0.001	0.33	5.8(1.8 to 9.9)	0.005
18.5–24.9	17.1(15.5 to 18.7)	15.6(14.4 to 16.7)	20.7(19.4 to 22.0)	21.6(20.2 to 23.0)	< 0.001		4.5(2.6 to 6.5)	< 0.001
25.0–29.9	18.7(17.0 to 20.4)	16.1(14.9 to 17.2)	20.3(18.9 to 21.6)	22.3(20.4 to 24.2)	< 0.001		3.6(1.2 to 6.0)	< 0.001
≥ 30.0	20.1(17.9 to 22.3)	19.2(17.2 to 21.2)	21.7(19.9 to 23.6)	23.1(20.5 to 25.8)	0.04		3.0(-0.3 to 6.4)	0.08

Values are % (95%CI). All calculations are weighted, accounting for the multistage cluster sampling design. *CI* Confidence interval, *PA* Physical activity, *MVPA* Moderate to vigorous physical activity

**Fig. 2:**
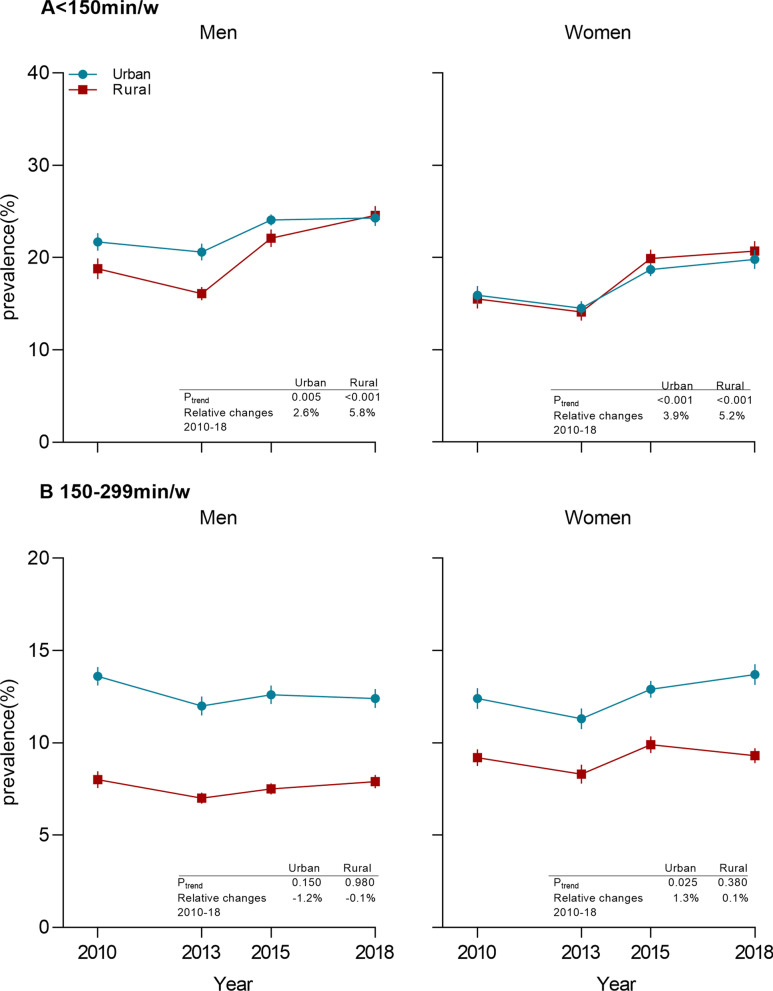
Trends in insufficient PA (<150 min/w) and percentages of adults undertaking 150-299 min/w of MVPA in China, 2010-18

The prevalence of insufficient PA increased in both rural (5.8 of the relative increase, *P* for trend < 0.001) and urban (2.6% of the relative increase, *P* for trend = 0.005) men in 2018 compared to 2010. A similar change was also observed in urban and rural women (*P* for trend < 0.001) (Fig. 2A). The increase in insufficient PA was observed among young adults aged 18–34 years in both rural (8.8% of the relative increase, *P* for change < 0.001) and urban (3.6% of the relative increase, *P* for change < 0.001). In addition, no significant change in insufficient PA was found except in the older age group of 65 years, however, all other age groups showed an upward trend (Appendix Table 1). Overall, no changes were observed in the percentages of adults undertaking 150–299 min/w of MVPA from 2010 to 2018 (*P* for trend = 0.23) (Appendix Table 2).

### Change in domain-specific no MVPA

**Fig. 3:**
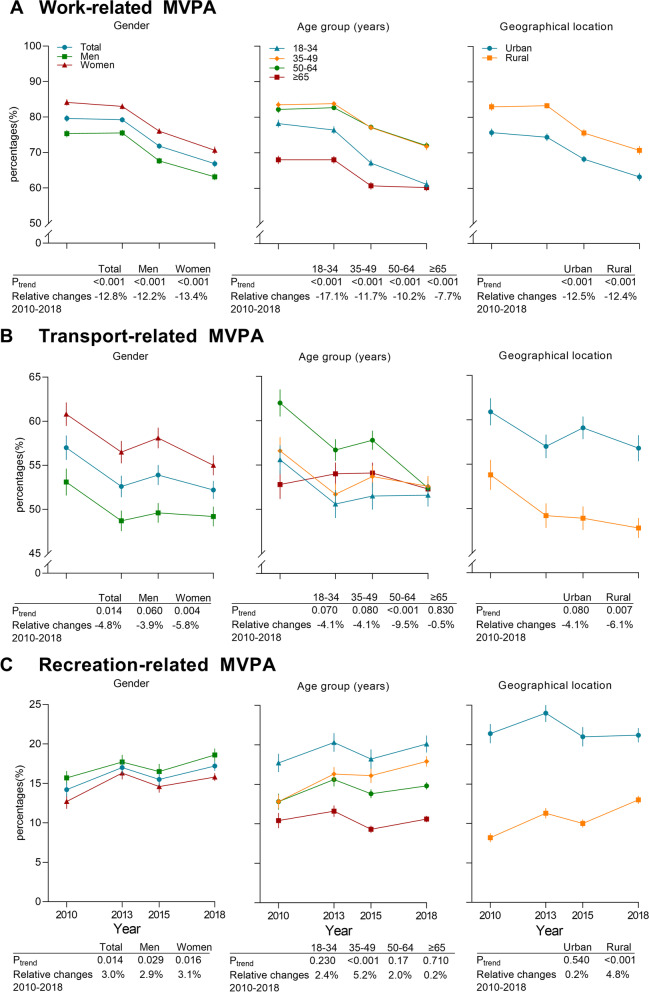
Trends in percentages of adults participating domain-specific MVPA in China, 2010-18

Regarding the change in participating in domain-specific MVPA, during the 8 years, the percentages of adults participating in work-related MVPA decreased most significantly, from 79.6% (77.8% to 81.5%) to 66.8% (64.9% to 68.7%) (*P* for trend < 0.001) (Fig. 3A), while the percentages of adults participating in recreation-related MVPA increased from 14.2% (12.5% to 15.9%) to 17.2% (16.0% to 18.4%) (*P* for trend = 0.014) (Fig. 3C). Participating in work-related MVPA was more prevalent among rural adults (70.7% [68.2% to 73.1%]) and women (70.6% [68.6% to 72.7%]) in 2018 (Fig. 3A). In contrast, the percentages of participating in recreation-related MVPA was higher among adults from urban areas (21.2% [19.5% to 23.0%]) and men (18.6% [17.0% to 20.2%] in 2018 (Fig. 3C). No change was observed in the percentages of adults participating in transport-related MVPA (Fig. 3B).

Appendix Table 4 shows the mean minutes of domain-specific MVPA per week. From 2010 to 2018, the mean minutes of work-related MVPA declined significantly in women from 922.5 min/wk (1276.7) to 817.1 min/wk (1385.6) (*P* for trend = 0.014) and in the youngest adults from 858.0 min/wk (1353.5) to 672.1 min/wk (1382.5) (*P* for trend < 0.001). A slight but significant increase was observed for minutes of transport-related MVPA overall and by characteristics (all *P* for trend < 0.001). For mean minutes of recreation-related MVPA, a significant increase was observed in men, women, adults aged less than 50 years, and those living in rural areas (all *P* for trend < 0.001). In addition, the relative contributions of domain-specific activity to total MVPA are shown in the appendix. (Appendix Table 5). There are similar research results in no intensity-specific MVPA among Chinese adults in our study. (Appendix Table 6).

## Discussion

Our study was the first large nationwide study assessing the long-term trend of insufficient PA in China. Data from four large consecutive and nationally representative surveys between 2010 to 2018 showed that insufficient PA among Chinese adults (both men and women) had increased since 2010. In urban, the prevalence of insufficient PA was still climbing between men and women, and there was also a rising trend in rural. Regarding the change in domain-specific MVPA, the prevalence of work-related and transport-related MVPA decreased most significantly, while recreation-related MVPA increased. In the stratified analysis, the percentage of work-related MVPA decreased significantly in both urban and rural areas, while the downward trend of transport-related MVPA and the upward trend of recreation-related MVPA were only significant in rural areas.

WHO has set a specific insufficient PA control goal, a 10% relative reduction in the prevalence of insufficient PA by 2025[2]. However, due to the lack of historical data, the Chinese government has not set a similar goal. This study showed that from 2010 to 2018, the overall insufficient PA prevalence in Chinese adults increased by 24.6%, slightly decreasing from 2010 to 2013. Guthold’s study showed that the global prevalence of insufficient PA was stable between 2001 (28.5%) and 2016 (27.5%), as well as divergent trends, were observed in different areas or countries [16]. This study showed that the overall increasing trend in China was consistent with western high-income countries, and both were in a relatively stable state. However, there was a certain rise in insufficient PA in our research. China’s current insufficient PA was lower than the global average and neighboring developed countries like Japan and South Korea, but higher than other East and Southeast Asia countries [16]. Such a position was comparable to China's economic development level globally. As for the decrease from 2010 to 2013, we considered the survey season as one of the possible reasons. In CCDRFS 2010, 2015, and 2018, more than 85% of participants were interviewed in late autumn and winter (e.g., November, December, and January). However, in CCDRFS 2013, this proportion was less than 30%. The previous study showed a seasonal effect as PA appeared to be highest in spring and summer [36]. In addition, a cohort study in Hong Kong, China, showed an inverted U-shaped association between temperature and outdoor PA, with both very low and very high temperatures associated with lower outdoor PA, and very low temperatures leading to greater PA reduction[37]. Cold weather might also be considered a barrier to PA in an American study [38, 39]. In our surveys, most participants were interviewed during late autumn and winter (from October to December) in 2010, 2015, and 2018. However, because of the expansion of CCDRFS completed in autumn of 2013, nearly 50% of participants were interviewed in early autumn (from August to October) in 2013 and about 35% of participants from new DSPs were interviewed in spring or early summer (from March to June) in 2014.

Insufficient PA levels varied considerably according to gender, age, geographical location, education, occupation, and BMI in China. We found that insufficient PA was higher for men than for women across all survey years and higher for those aged 65 or older and 18–34 than those aged 35–64. Previous studies showed a general increase in insufficient PA with advancing age in both men and women [40]. From 2010 to 2015, insufficient PA of Chinese people aged 65 or older still was higher than those aged 18–64, according to our study. However, it was worth noting that the prevalence of insufficient PA in adults aged 18–34 surpassed that of those aged 65 or older in 2018. About 50% of adults over 65 in the “Aging in Chianti” study experienced no change in average PA, and about 13% reported an increase in PA over a 3-year follow-up [41]. Therefore, the trend of insufficient PA in the elderly still needs further research. Additionally, we found that the prevalence of insufficient PA among urban residents was higher than that of rural residents, but the upward trend was more pronounced in rural areas, which had surpassed urban areas by 2018. Likewise, a previous research report also showed that insufficient PA was higher in urban residents than among men and women rural residents [42]. The difference in low MVPA also exists among people with different levels of education in our study. Similarly, a Japanese study found that men with high educational qualifications had significantly lower PA than those with lower educational qualifications [43]. In occupational groups, our study observed that unemployed people and students had the highest prevalence of insufficient PA. An Armenia study also found that unemployed people were less engaged in PA and less likely to meet WHO recommendations on PA [44]. Interestingly, the present study found that Chinese adults with a BMI < 18.5 kg/m^2^ had a less prevalence of PA, and others showed increased PA as their BMI increased. In accordance with the present results, previous studies have reported the lowest PA in underweight Korean adults [45]. The recent study also showed that the prevalence of insufficient PA in normal-weight people was lower than the obese individuals, and the prevalence of insufficient PA was increasing in all BMI categories [45]. Insufficient PA increases the prevalence of chronic diseases such as obesity, hypertension, and diabetes, affecting the balance of energy metabolism, muscle function, and peripheral insulin resistance [46, 47]. According to our research results, the current situation of insufficient PA in China is not optimistic and will seriously endanger the health of residents.

Besides the decline in total PA, the study also showed that fewer adults participated in work-related MVPA and that the amount of time spent on work-related MVPA decreased. Between 2004 and 2011, a study also suggested that for both adult men and women in China, work and domestic PA levels fell by nearly half, and the decline was more pronounced for women from 2004 to 2011 [48]. A study covering 104 countries in the world reported that China also has such a trend, but work-related PA was higher in low-income countries [18]. Therefore, socio-economic factors also have an important impact on PA. In the past decades, China's industrial structure has undergone significant changes. The contribution rate of the Tertiary Industry (mainly the service industry) to Gross Domestic Product (GDP) has continued to increase since the early 1980s and has surpassed both Primary (agricultural, 8.9%) and Secondary (manufacturing, 44.2%) industries since 2013 and accelerated in the following years. Meanwhile, the employed persons in primary and secondary industries continued to decline with a significant increase in the tertiary industry [49]. This may be an essential factor leading to the decline in work-related PA among Chinese residents. Moreover, the current study has shown that the mean contribution of work-related MVPA was the most in total MVPA. These results agreed with those obtained by a global study about levels of domain-specific PA [18]. We also found that the mean contribution of work-related MVPA was declining from 2010 to 2018, while the mean contribution of recreation-related was rising. This phenomenon may be a primary factor in changing domain-specific PA in China. Furthermore, a Brazilian study showed that work-related PA has a higher contribution to poorer groups [50]. In our analysis, work-related PA was significantly higher among individuals living in rural residences than those living in urban residences, confirming the conclusion from the side. Additionally, research suggested that more work-related PA was associated with poorer physical health and a greater risk of early death [51]. As such, work-related PA might not have the same benefits to health and well-being as other domain-specific, particularly recreation. Despite this, global PA guidelines encourage adults to be active during any life domain, including leisure time, transport, occupational, and household chores PA [52]. After all, sedentary behaviors at work can also lead to health risks [53]. An increasing trend in recreation-related MVPA was found in our research, which means more adults participated in the leisure time PA. Andreas et al. found higher recreation-related PA associated with reduced cardiovascular disease and all-cause mortality risk, while higher work-related PA was associated with increased risks, independent of each other [53]. Thus, the current trend of recreation-related PA in China is beneficial to the health of residents.

The world and nations have already formulated some PA policies. For example, the “Global Recommendations on Physical Activity for Health” published by WHO was to guide decision-makers at the national and regional levels [54]. Whereafter, China also published “Several Opinions on Accelerating the Development of the Sports Industry and Promoting Sports Consumption” in 2014 [55] and the Outline of the “Healthy China 2030” Plan in 2016 [56], to make fitness for all people a national strategy, and improve people's physical fitness. Our study found that the trend of recreation-related PA was relatively optimistic, which may be attributed to the above policies. However, it is worth noting that the total insufficient PA was increasing in China, especially with the obvious decline in work-related PA.

Our study reported the trend of insufficient PA from 2010 to 2018, which could not reflect the change caused by the global pandemic in 2019. The global pandemic occurred and disrupted daily behavior patterns possibly. Results of a meta-analysis showed that a downward trend in PA was observed in adults from Italy, the United States, China, Japan, Spain, Singapore, and South Korea during the global pandemic[57]. A study of New Zealand during the pandemic also showed a significant reduction in participation in PA during the lockdown from June 2020 to April 2021, which may have an impact on subsequent health outcomes and related intervention programs[58]. Until now, no studies addressing the impact of the pandemic on PA among adults nationwide in China have been reported. We look forward to the next round of CCDRFS and future analysis of changes in insufficient PA after the global pandemic.

### Strengths and limitations

Our study used multiple large nationally representative health survey data, and a total of 645 903 adults were included from 2010 to 2018, measured using the GPAQ, to describe PA trends in China. Also, the response rates in all four surveys were high, and the sampling ensured that the samples were representative of the general population in China. However, our study also has limitations. First, the PA in this study was self-reported, which was not as accurate as measured by objective instruments. When comparing the GPAQ with accelerometers and pedometers, it was found that the GPAQ data had poor concurrent validity[31]. In our research, the GPAQ was conducted in face-to-face interviews, which have excellent reliability and validity and have been widely used worldwide [25, 59], and the interviewers have received professional training. The phenotypic agreement was generally good between subjective and objective measures of insufficient PA [54, 55]. Also, it is hard to use accelerometers and other instruments to measure attitude activities in large-scale epidemiological investigations. Second, we adopted a replacement method to obtain a sufficient sample size. This sampling method was reasonable, considering that selecting replacement households when sampling was common in epidemiology studies. It was also applied in previous studies in China [60, 61] and other countries [62, 63]. The characteristics of the participants without replacement and after replacement in each CCDRFS survey were generally comparable. Therefore, the impact of replacement on the research results might be ignored. Finally, this study only described the trend of insufficient PA from 2010 to 2018 and did not explore the relationship between insufficient PA and chronic diseases. In the future, we will further conduct in-depth research on the relationship between insufficient PA and chronic non-communicable diseases.

## Conclusions

In conclusion, although the situation of recreation-related MVPA had gradually improved in the past nine years, work-related and transport-related MVPA continued to decline, resulting in an increase in overall insufficient PA among Chinese adults from 2010 to 2018. Our research provides strong new evidence for the evolving insufficient PA epidemic among adults in China. PA is needed to be integrated into different areas of people's daily work, transport, and recreation. Moreover, the implementation of increasing PA will lower the incidence of chronic non-communicable diseases (NCDs), cancer, and other diseases, thus reducing mortality. Policies that support decreasing insufficient PA can also provide benefits for health, wealth, employment, and environmental sustainability. China will have a hopeful future only if the country implements strong actions against insufficient PA.

## Supplementary Information

**Additional file 1:  Appendix2.** Data collection of CCDRFS 2010-18. **Appendix 3.** Analysis plan. **Appendix 4.**Global Physical Activity Questionnaire. **Appendix 5.** List of the typical physical activities. **Appendix figure 1.** Map of China Chronic Disease and Risk Factor Surveillance (CCDRFS) Sites. **Appendix figure 2.** Percentages of participants interviewed by month and survey. **Appendix table 1.** Trends in insufficient physical activity in urban and rural adults in China, 2010-18.**Appendix table 2.** Trends in adults undertaking 150-299 min/week of MVPA in China, 2010 -18. **Appendix table 3.** Trends in percentages of adults participating in domain-specific MVPA in China, 2010-18.**Appendix table 4.** Trends in mean min/week of domain-specific MVPA among adults in China, 2010-18. **Appendix table 5.** Mean domain-specific relative contribution to total MVPA among adults in China, 2010-18. **Appendix table 6.** Trends in percentages of adults without intensity-specific MVPA in China, 2010-18.

## Data Availability

Individual participant data in our study will not be made available publicly. For further detailed data access policy and procedure, please contact jianceshi@ncncd.chinacdc.cn.

## Abbreviations

PA: Physical activity
NCDs: Non-communicable diseases
MVPA: Moderate- to vigorous-intensity physical activity
CCDRFS: China Chronic Disease and Risk Factor Surveillance
BMI: Body mass index
China CDC: Chinese Center for Disease Control and Prevention
DSPs: Disease Surveillance Points
PPS: Proportional to population size
SRS: Systematic random sampling
NCNCD: National Center for Chronic and Noncommunicable Disease Control and Prevention
GPAQ: Global Physical Activity Questionnaire
WHO: World Health Organization
GDP: Gross Domestic Product
