# Supplementary material for: Trends in insufficient physical activity among adults in China 2010–18: a population-based study
Source: Int J Behav Nutr Phys Act. 2023 Jul 17;20:87. doi: 10.1186/s12966-023-01470-w (PMC10351178; doi:10.1186/s12966-023-01470-w)
Supplement: Supplementary file 1 — Additional file 1: Appendix2. Data collection of CCDRFS 2010-18. Appendix 3. Analysis plan. Appendix 4.Global Physical Activity Questionnaire. Appendix 5. List of the typical physical activities. Appendix figure 1. Map of China Chronic Disease and Risk Factor Surveillance (CCDRFS) Sites. Appendix figure 2. Percentages of participants interviewed by month and survey. Appendix table 1. Trends in insufficient physical activity in urban and rural adults in China, 2010-18.Appendix table 2. Trends in adults undertaking 150-299 min/week of MVPA in China, 2010 -18. Appendix table 3. Trends in percentages of adults participating in domain-specific MVPA in China, 2010-18.Appendix table 4. Trends in mean min/week of domain-specific MVPA among adults in China, 2010-18. Appendix table 5. Mean domain-specific relative contribution to total MVPA among adults in China, 2010-18. Appendix table 6. Trends in percentages of adults without intensity-specific MVPA in China, 2010-18. [file 12966_2023_1470_MOESM1_ESM.docx]

**Appendix**

**Trends in insufficient physical activity among adults in** **China 2010-18: population-based study**

**Journal name:** International Journal of Behavioral Nutrition and Physical Activity

**Authors**

Mei Zhang, PhD^1†^; Yanan Ma, PhD^2,3†^; Xili Xie, BS^2^; Ming Sun, BS^2^; Zhengjing Huang, BS^1^; Zhenping Zhao, MS^1^; Xiao Zhang, PhD^1^; Chun Li, MS^1^; Xingxing Gao, BS^1^; Jing Wu, PhD^1#^; Limin Wang, MPH^1*^; Maigeng Zhou^1*^, PhD; Deliang Wen, PhD^3*^

^†^These authors contributed equally as first co-authors.

^#^Senior author.

^*^These authors contributed equally as corresponding co-authors.

**Author affiliations:**

1. National Centre for Chronic and Non-communicable Disease Control and Prevention, Chinese Centre for Disease Control and Prevention, 27 Nanwei Road, Xicheng District, Beijing, 100050, China

2. Department of Biostatistics and Epidemiology, School of Public Health, China Medical University, No.77 Puhe Road, Shenyang, Liaoning Province 110122, China

3. Health Sciences Institute, China Medical University, No.77 Puhe Road, Shenyang, Liaoning Province 110122, China; Liaoning Key Laboratory of Obesity and Glucose/Lipid Associated Metabolic Diseases, China Medical University, Shenyang, Liaoning Province 110122, PR China

**The Corresponding Authors:**

1. Prof. Limin Wang

National Centre for Chronic and Non-communicable Disease Control and Prevention, Chinese Centre for Disease Control and Prevention, Beijing, China 100050

E-mail: [wanglimin@ncncd.chinacdc.cn](mailto:wanglimin@ncncd.chinacdc.cn)

Tel: +8610 83136482

Fax: +8610 63042350

1. Maigeng Zhou

National Centre for Chronic and Non-communicable Disease Control and Prevention, Chinese Centre for Disease Control and Prevention, Beijing, China 100050

E-mail: [zhoumaigeng@ncncd.chinacdc.cn](mailto:zhoumaigeng@ncncd.chinacdc.cn)

Tel: +8610 63041471

Fax: +8610 63042350

1. Deliang Wen

Health Sciences Institute, China Medical University, No.77 Puhe Road, Shenyang, Liaoning Province 110122, China; Liaoning Key Laboratory of Obesity and Glucose/Lipid Associated Metabolic Diseases, China Medical University, Shenyang, Liaoning Province 110122, PR China

E-mail: dlwen@cmu.edu.cn

Tel: +86 024-31939003

Fax: +86 024-31939003

Contents

[**Appendix 1. Sample design of CCDRFS 2010-18.** 4](#_Toc140232188)

[**Appendix 2. Data collection of CCDRFS 2010-18.** 8](#_Toc140232189)

[**Appendix 3. Analysis plan.** 10](#_Toc140232190)

[**Appendix 4. Global Physical Ativity Questionnaire** 12](#_Toc140232191)

[**Appendix 5. List of the typical physical activities** 14](#_Toc140232192)

[**Appendix figure 1. Map of China Chronic Disease and Risk Factor Surveillance (CCDRFS) Sites** 15](#_Toc140232193)

[**Appendix figure 2. Percentages of participants interviewed by month and survey** 15](#_Toc140232194)

[**Appendix table 1. Trends in insufficient physical activity in urban and rural adults in China, 2010-18** 16](#_Toc140232195)

[**Appendix table 2. Trends in adults undertaking 150-299 min/week of MVPA in China, 2010 -18** 18](#_Toc140232196)

[**Appendix table 3. Trends in percentages of adults participating in domain-specific MVPA in China, 2010-18** 19](#_Toc140232197)

[**Appendix table 4. Trends in mean min/week of domain-specific MVPA among adults in China, 2010-18** 20](#_Toc140232198)

[**Appendix table 5. Mean domain-specific relative contribution to total MVPA among adults in China, 2010-18** 22](#_Toc140232199)

[**Appendix table 6. Trends in percentages of adults without intensity-specific MVPA in China, 2010-18** 23](#_Toc140232200)

# **Appendix 1. Sample design of CCDRFS 2010-18.**

**Overview**

The sample of CCDRFS was obtained from China’s national Disease Surveillance Points system (DSPs) (1). One DSP unit covers a rural county or an urban district. The DSPs were established during the early1980s, using multi-stage stratified sampling with probability proportional to size (PPS) to ensure the representativeness of the national population of mainland China. Following further expansion and sampling enhancement from the 2013 survey, the selected DSPs were also represented at the provincial level. CCDRFS is planned and administrated by the National Centre for Chronic and Non-communicable Disease Control and Prevention (NCNCD) of the Chinese Centre for Disease Control and Prevention (China CDC).

**Disease Surveillance Points system (DSPs)**

The DSPs were piloted in 1978 and fully established in the early 1980s, covering initially 71 counties/districts, expanding to 145 in 1989 and 161 DSPs in 2004 (covering a population of 73 million) from 31 provinces (autonomous regions, and municipalities) to accommodate the societal and economic development during this period. For each expansion, the population characteristics of the selected DSPs were compared to the census population to ensure its national representativeness(2, 3). In 2013, the Chinese government combined the DSPs system with the national vital registration system to form a new DSPs system, also named an integrated national mortality surveillance system, which increased the DSPs areas from 161 to 605. The DSPs system currently covers 324 million Chinese adults (24% of the Chinese population) in the mainland of China (4).

**Sampling procedure**

Embedded within the DSPs, the CCDRFS used stratified multi-stage cluster sampling to generate a nationally representative sample for each survey.

In the first stage, half of the DSPs were selected as primary sampling units (PSUs) using stratified sampling (in the surveys of 2013, 2015, and 2018), or all DSPs were selected as PSUs (in the 2010 survey). Since the 2013 survey, following the expansion of the DSPs, 298 DSPs were selected from all 605 DSPs to generate a sample representative of both the national and provincial population in the 31 provinces (autonomous regions, municipalities) of mainland China. The sampling of DSPs was done centrally by the NCNCD.

Then, within each selected DSP, the following steps were followed:

1. Townships (rural) or subdistricts (urban) were selected with the Proportional Probability Sampling (PPS) method (survey 2010 and 2013) or Systematic Random Sampling (SRS, survey 2015 and 2018) in each selected DSP.
2. Within the selected townships and subdistricts, villages (rural) or residential areas (urban) were selected with PPS (survey 2010 and 2013) or SRS (survey 2015 and 2018).
3. Each selected village or residential area was divided into groups of about 50 households (60 households since 2015), based on existing villager/resident groups in the village or residential area. One group was selected with simple random sampling. Before 2015, all households in the group selected were invited to participate in the survey. Since 2015, 45 households were randomly selected from the selected group (60 households).
4. Within each selected household, in the surveys of 2010 and 2013, the Kish method was used to select one eligible adult. In the 2015 and 2018 surveys, all eligible adults in the household were invited.

Eligibility criteria include:

- 1. Aged 18 years or older;
  2. Having lived at the address for more than six months in the past 12 months;
  3. Not pregnant;
  4. With no severe health condition or illness that would prevent the participant from participating, including intellectual disability or language disorder.

1. In a household, if the selected eligible adult (the surveys of 2010 and 2013) or more than half of the family’s eligible members (the 2015 and 2018 surveys) refused to participate or were not reachable, a replacement was selected using the following principles:

a) the whole household should be replaced.

b) a household in the same residential quarter would be chosen as the replacement if possible.

c) the replacement candidate household should have a family structure similar to the original household.

For four surveys, 6% of participants were replaced. The characteristics of samples without replacement and after replacement were shown in Appendix table 11.

For surveys in 2010 and 2013, the sampling within selected DSPs, as described above, was conducted by the NCNCD centrally; since the 2015 survey, the sampling was performed by the provincial CDCs and approved by the NCNCD.

**Sample size calculation**

The sample size of each round of CCDRFS was calculated using N =

where *u* = 1.96 (corresponding to 95% confidence level), *p* is the expected prevalence of the factor studied for calculation, *deff* is designed effects (1.5 to 3.5), *r* is a relative error (20%), and *d* is margin of error (*d* = *r* × *p*). The CCDRFS surveys used obesity prevalence (2010) or diabetes prevalence (2013/2015/2018) from previous national surveys (8.0%-10.4%). The table below shows the calculated target sample sizes in each survey. Because the previous physical inactivity prevalence was higher than the prevalence of diabetes in each survey year, the calculated sample size could accurately estimate the insufficient physical activity prevalence, overall and by subgroup.

| Survey | Indicators | Prevalence | Target sample size |
| --- | --- | --- | --- |
| 2010 | Obesity | 8.0% in 2007 | 96 870 |
| 2013 | Diabetes | 9.7% in 2010 | 181 000 |
| 2015 | Diabetes | 10.4% in 2013 | 184 773 |
| 2018 | Diabetes | 10.4% in 2013 | 181 059 |

**Sample weights**

Across all CCDRFS surveys, we developed sample weights to account for multi-stage sampling design, and post-stratification, for the 2015 and 2018 surveys only, non-response within the household as all members were selected. For an individual in the sample, his/her sample weights were developed as follows.

1. Base weights for multi-stage design (W_design_)

W_design_ = W_d1_ × W_d2_ × W_d3_ × W_d4_ × W_d5_ × W_d6_

- W_d1_ is the total number of counties (rural) or districts (urban) in the stratum divided by the number of selected DSPs (PSU) in the stratum where the individual was from;
- W_d2_ is the total number of townships (rural) or subdistricts (urban) in the PSU where the individual belongs divided by the number of selected townships or subdistricts;
- W_d3_ is the total number of villages (rural) or residential areas (urban) in the township or subdistrict where the individual was from, divided by the number of selected villages or residential areas;
- W_d4_ is the total number of groups in the village or residential area where the individual was from;
- W_d5_ is the total number of households in the group where the individual belonged divided by the number of selected households in the group where the individual was from;
- W_d6_ is the total number of eligible adults in the household where the individual was from (2013 or earlier) or 1 (since 2015).

1. Non-response weights (W_nr_) in the 2015 and 2018 surveys

W_nr_ = the number of eligible adults in the household where the individual was divided by the number of participating adults in the household.

1. Post-stratification weights (W_ps_)

Stratifications included: province (31 levels), urban or rural (2 levels), sex (2 levels), and age group (13 levels: 18-24, 25-29, 30-34, 35-39, 40-44, 45-49, 50-54, 55-59, 60-64, 65-69, 70-74, 75-79, 80+). The 2010 census population was also stratified in the same way. In k^th^ stratum, the post-stratification weights (Wps,k) are:

$$Wps,k= \frac{Population in the k^{\mathrm{th}} stratum of the 2010 census population}{Sum of Wdesign\times Wnr for all individuals in the k^{\mathrm{th}}\mathrm{stratum}}$$

# **Appendix 2. Data collection of CCDRFS 2010-18.**

**Overview**

The CCDRFS fieldwork was coordinated by the NCNCD and implemented by the local CDCs. All fieldwork staff received mandatory training provided by certified instructors from the NCNCD and hosted by provincial CDCs.

The fieldwork in each round of surveys started in August. In most rounds (2010, 2015, and 2018), most interviews were finished in the same year, and the remaining visits were completed by January (2010) and June (2015 and 2018) of the following year. For CCDRFS 2013, most interviews of 161 DSPs that have conducted fieldwork for CCDRFS 2010 were finished in the early autumn of 2013. However, since the expansion of the DSPs system was completed in 2013, most interviews of newly added CCDRFS surveillance districts or counties were surveyed in the spring of the following year (**Appendix figure 2**).

Trained interviewers from local CDCs carried out face-to-face interviews, physical measurements, and biochemical sample collection. The face-to-face interview was conducted during a home visit. All subjects meeting the inclusion criteria were invited to a community health center to attend a physical measurement and biochemical sample collection session. Subjects who did not complete their interviews during home visits also completed their interviews in the health center.

**Home visits**

During the home visit, the head of the household or an adult who knew well the household details (e.g., economic and environmental information) was interviewed first using the household questionnaire that assessed the eligibility of all household members. Then, every eligible subject was given an individual questionnaire that covered demographic characteristics, lifestyle factors (e.g., smoking and cessation, diet behavior, insufficient physical activity, alcohol consumption), and history of chronic diseases. At the end of the home visit, the investigator gave each subject an appointment letter, instructing the location, time, and precautions of the physical measurements.

**Physical measurements**

All subjects were invited to the local community health care station or village clinic to attend a physical measurement session. Subjects who did not complete their interviews during home visits also completed their interviews in the health care station. Weight, height, waist circumference, and blood pressure were measured using a standard protocol.

**Data collection and management**

From 2010 to 2013, all data were recorded on paper questionnaires first, and then entered into the computer via a bespoke data management system and delivered to the NCNCD via email (2010) or an online data transfer system (2013). All the data entry and processing were done by the local CDCs. Since 2015, NCNCD developed an integrated platform consisting of a tablet-assisted interview system and an online information management system, which was adopted by all local CDCs. The data from the questionnaire and physical measurements were collected electronically using the tablet-assisted system. The internet-based information management system was used to generate samples (as described in **Appendix 1**), on-site identity confirmation, quality control, physical examination reports, and data download.

# **Appendix 3. Analysis plan.**

The analysis aims to describe the trends from 2010 to 2018 in the prevalence of insufficient physical activity in China overall and by subgroups of the population.

**Study participants**

Men and women aged 18 years old and above.

**Primary outcomes**

Prevalence of physical inactivity, including moderate to vigorous insufficient physical activity (MVPA) <150 minutes/week.

**Secondary outcomes**

1. Percentage of adults undertaking 150-299 minutes/week of MVPA.
2. Percentage of adults participating in domain-specific MVPA, including work-, transport-, and recreation-related PA.
3. Domain-specific mean minutes of MVPA per week.
4. Percentage of adults not involved in intensity-specific MVPA, including vigorous, moderate, and both vigorous and moderate PA.

**Stratification**

1. **Year**: 2010, 2013, 2015, 2018.
2. **Sex**: men, women.
3. **Age**: 18-34, 35-49, 50-64, ≥65 years.
4. **Geographic location**: rural, urban.
5. **Education**: secondary school or less, high school, college or above.
6. **Occupation**: agriculture-related work, other manual work, non-manual work (e.g., professionals, public servants, housewives), unemployed /students, retired).
7. **BMI:** (<18.5kg/m^2^,18.5~24.9kg/m^2^, 25.0~29.9kg/m^2^, ≥30kg/m^2^).

**Data cleaning**

The following data cleaning criteria recommended by the analysis guide were also used in our analyses:

1. Each participant included in the analyses must have a valid response for at least one domain and have no invalid responses for any domains.
2. If a participant reports implausible values (e.g., >7 days in any days column), remove the case from all analyses.
3. If a participant has inconsistent answers (e.g., 0 days, but values >0 in the corresponding time variables), remove the case from all analyses.
4. If one whole "sub-domain" (vigorous work, moderate work, transport, vigorous recreation, or moderate recreation activity) has missing values, but the other "sub-domains" are valid, including the case in the analysis, assuming no activity (0 days, 0 times) for this "sub-domain".
5. If the time of one whole "sub-domain" is more than 960 minutes per week, remove the case from all analyses.
6. Individuals with missing demographic information or insufficient physical activity-related variables are excluded from the analyses.

**Statistics**

As recommended by the Global Insufficient physical activity Questionnaire (GPAQ) Analysis Guide, for the calculation of a person's overall MVPA minutes using GPAQ data, the following process is used:

| Domain-specific minutes | Equation |
| --- | --- |
| Work-related MVPA | Minutes of vigorous PA/day×days/week×2+ Minutes of moderate PA/day×days/week |
| Transport-related PA | Minutes of PA/day×days/week |
| Recreation-related MVPA | Minutes of vigorous PA/day×days/week×2+ Minutes of moderate PA/day×days/week |

Total MVPA minutes= Work-related MVPA+ Transport-related PA+ Recreation-related MVPA

Outcome (primary and secondary) levels, overall and in groups as defined in ‘Stratification’.

- SAS is used to account for clustering, stratification, and sample weights in the sample design.
- For the prevalence and means, standard error and 95% confidence interval are estimated using *proc* *surveylogistic* procedure and *proc surveymeans* procedure in SAS, using Taylor series linearisation and accounting for finite population correction (5,6).

**References**

1. Yang G, Hu J, Rao KQ, Ma J, Rao C, Lopez AD. Mortality registration and surveillance in China: History, current situation and challenges. Popul Health Metr. 2005;3(1):3.

2. Yang G. [Selection of DSP points in second stage and their presentation]. Zhonghua Liu Xing Bing Xue Za Zhi. 1992;13(4):197-201.

3. Zhou MG, Jiang Y, Huang ZJ, Wu F. Adjustment and representativeness evaluation of national disease surveillance points system. Dis Surveill. 2010;25(3):239-44.

4. Liu S, Wu X, Lopez AD, et al. An integrated national mortality surveillance system for death registration and mortality surveillance, China [J]. Bull World Health Organ, 2016, 94(1): 46-57.

5. SAS Institute Inc. SAS/STAT® 14.2 User’s Guide. The SURVEYLOGISTIC Procedure; 2016.

6. SAS Institute Inc. SAS/STAT® 14.3 User’s Guide. The SURVEYMEANS Procedure; 2017.

# **Appendix 4. Global Physical Ativity Questionnaire**

| **CORE: Insufficient physical activity** | | | |
| --- | --- | --- | --- |
| Next, I am going to ask you about the time you spend doing different types of insufficient physical activity in a typical week. Please answer these questions even if you do not consider yourself to be a physically active person.  Think first about the time you spend doing work. Think of work as the things that you have to do such as paid or unpaid work, study/training, household chores, harvesting food/crops, fishing or hunting for food, and seeking employment. *[Insert other examples if needed].* In answering the following questions 'vigorous-intensity activities' are activities that require hard physical effort and cause large increases in breathing or heart rate, and 'moderate-intensity activities' are activities that require moderate physical effort and cause small increases in breathing or heart rate. | | | |
| **Question** | **Response** | | **Code** |
| **Work** | | | |
| Does your work involve vigorous-intensity activity that causes large increases in breathing or heart rate like *[carrying or lifting* *heavy loads, digging, or construction work]* for at least 10 minutes continuously?  *[INSERT EXAMPLES] (USE SHOWCARD)* | Yes | 1 | 0B*E1* |
|  | No | 2  *If No, go to E 4* |  |
| In a typical week, how many days do you do vigorous-intensity activities as part of your work? | Number of days | └─┘ | 1B*E2* |
| How much time do you spend doing vigorous-intensity activities at work on a typical day? | Hours: minutes | └─┴─┘: └─┴─┘  hr mins | E3 (a-b) |
| Does your work involve moderate-intensity activity, that causes small increases in breathing or heart rate such as brisk walking *[or carrying light loads]* for at least 10 minutes continuously?  *[INSERT EXAMPLES] (USE SHOWCARD)* | Yes | 1 | E4 |
|  | No | 2 *If No, go to E 7* |  |
| In a typical week, how many days do you do moderate-intensity activities as part of your work? | Number of days | └─┘ | E5 |
| How much time do you spend doing moderate-intensity activities at work on a typical day? | Hours: minutes | └─┴─┘: └─┴─┘  hrs mins | E6 (a-b) |
| **Travel to and from places** | | | |
| The following questions exclude the physical activities at work that you have already mentioned.  Now I would like to ask you about the usual way you travel to and from places. For example, to work, for shopping, to market, to the place of worship. *[Insert other examples if needed]* | | | |
| Do you walk or use a bicycle *(pedal cycle)* for at least 10 minutes to get to and from places? | Yes | 1 | E7 |
|  | No | 2  *If No, go to E 10* |  |
| In a typical week, on how many days do you walk or bicycle for at least 10 minutes continuously to get to and from places? | Number of days | └─┘ | E8 |
| How much time do you spend walking or bicycling for travel on a typical day? | Hours: minutes | └─┴─┘: └─┴─┘  hr mins | E9 (a-b) |

| **CORE: Insufficient physical activity, Continued** | | | |
| --- | --- | --- | --- |
| **Question** | **Response** | | **Code** |
| **Recreational activities** | | | |
| The next questions exclude the work and transport activities that you have already mentioned.  Now I would like to ask you about sports, fitness and recreational activities (leisure), *[Insert relevant terms]*. | | | |
| Do you do any vigorous-intensity sports, fitness, or recreational *(leisure)* activities that cause large increases in breathing or heart rate like *[running or football]*  for at least 10 minutes continuously?  *[INSERT EXAMPLES] (USE SHOWCARD)* | Yes | 1 | E10 |
|  | No | 2  *If No, go to E 13* |  |
| In a typical week, how many days do you do vigorous-intensity sports, fitness, or recreational *(leisure)* activities? | Number of days | └─┘ | E11 |
| How much time do you spend doing vigorous-intensity sports, fitness, or recreational activities on a typical day? | Hours: minutes | └─┴─┘: └─┴─┘  hrs mins | E12  (a-b) |
| Do you do any moderate-intensity sports, fitness, or recreational *(leisure)* activities that cause a small increase in breathing or heart rate such as brisk walking*, [cycling, swimming, or volleyball]* for at least 10 minutes continuously?  *[INSERT EXAMPLES] (USE SHOWCARD)* | Yes | 1 | E13 |
|  | No | 2  *If No, go to E 16* |  |
| In a typical week, how many days do you do moderate-intensity sports, fitness, or recreational *(leisure)* activities? | Number of days | └─┘ | E14 |
| How much time do you spend doing moderate-intensity sports, fitness, or recreational *(leisure)* activities on a typical day? | Hours: minutes | └─┴─┘: └─┴─┘  hrs mins | E15 (a-b) |

# **Appendix 5. List of the typical physical activities**

| **WORK RELATED INSUFFICIENT PHYSICAL ACTIVITY** | | **RECREATION RELATED INSUFFICIENT PHYSICAL ACTIVITY** | |
| --- | --- | --- | --- |
| **MODERATE**  **Intensity Activities**  Makes you breathe somewhat harder than normal | **VIGOROUS**  **Intensity Activities**  Makes you breathe much harder than normal | **MODERATE**  **Intensity Activities**  Makes you breathe somewhat harder than normal | **VIGOROUS**  **Intensity Activities**  Makes you breathe much harder than normal |
| **Examples:**   - Working on the assembly line (industry) - Cleaning (vacuuming, mopping, polishing, scrubbing, sweeping, ironing) - Washing (beating and brushing carpets, wringing clothes (by hand) - Gardening - Milking cows (by hand) - Planting and harvesting crops - Digging dry soil (with spade) - Weaving - Woodwork (chiseling, sawing softwood) - Mixing cement (with shovel) - Labouring (pushing loaded wheelbarrow, operating jackhammer) - Walking with load on head - Drawing water - Tending animals | **Examples:**   - Forestry (cutting, chopping, carrying wood) - Sawing hardwood - Ploughing - Cutting crops (sugar cane) - Gardening (digging) - Grinding (with pestle) - Laboring (shoveling sand) - Loading furniture (stoves, fridge) - Instructing spinning (fitness) - Instructing sports aerobics - Sorting postal parcels (fast pace) - Cycle rickshaw driving | **Examples:**   - Cycling - Jogging - Dancing - Horse-riding - Tai chi - Yoga - Pilates - Low-impact aerobics - Cricket | **Examples**   - Table tennis - Soccer - Rugby - Tennis - High-impact aerobics - Aqua aerobics - Ballet dancing - Fast swimming |


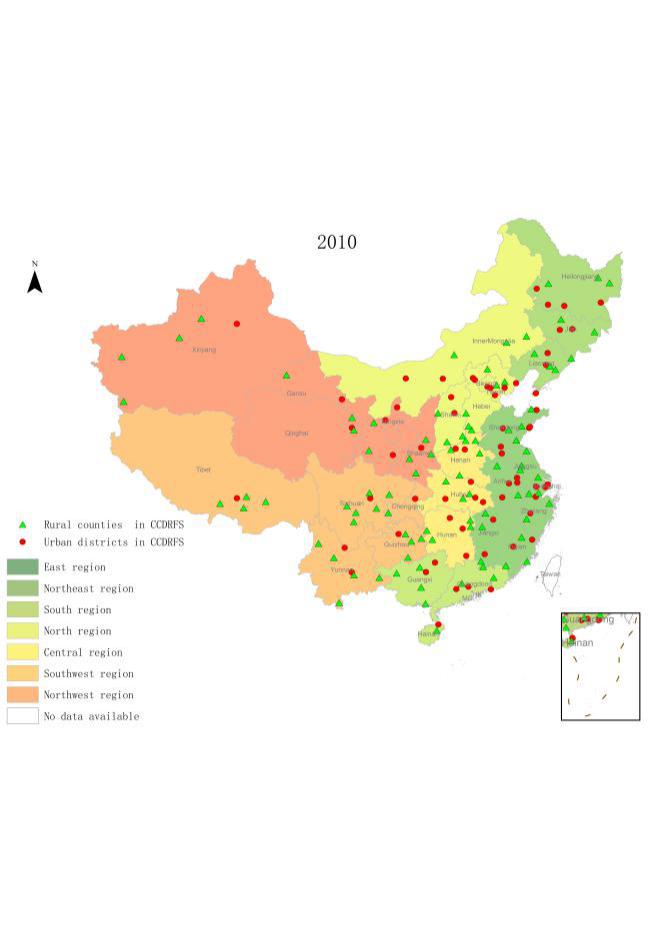


2010


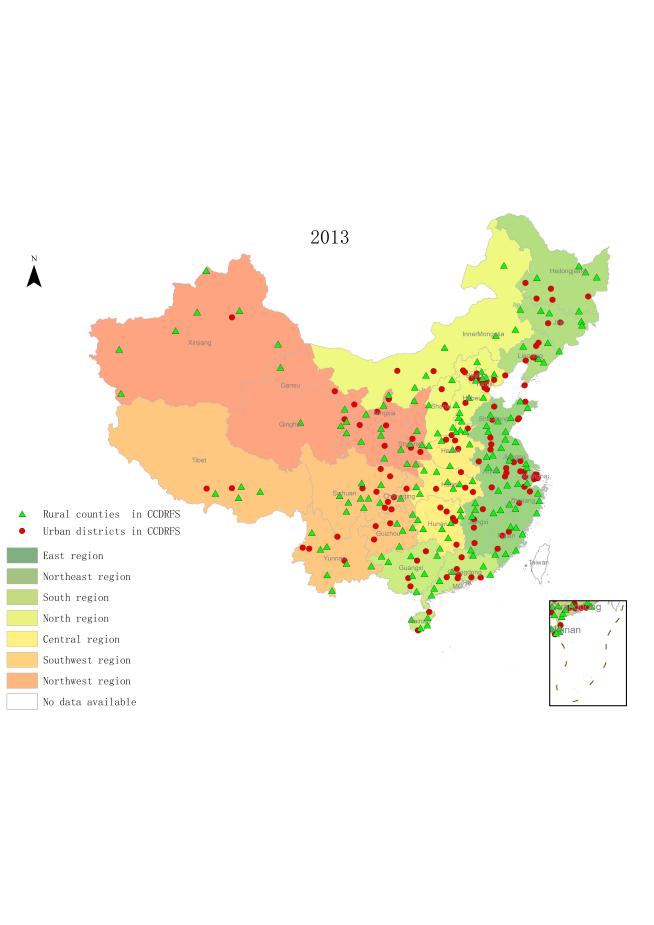


2013-2018

MO: Macao; HK: Hongkong

# **Appendix figure 1. Map of China Chronic Disease and Risk Factor Surveillance (CCDRFS) Sites**


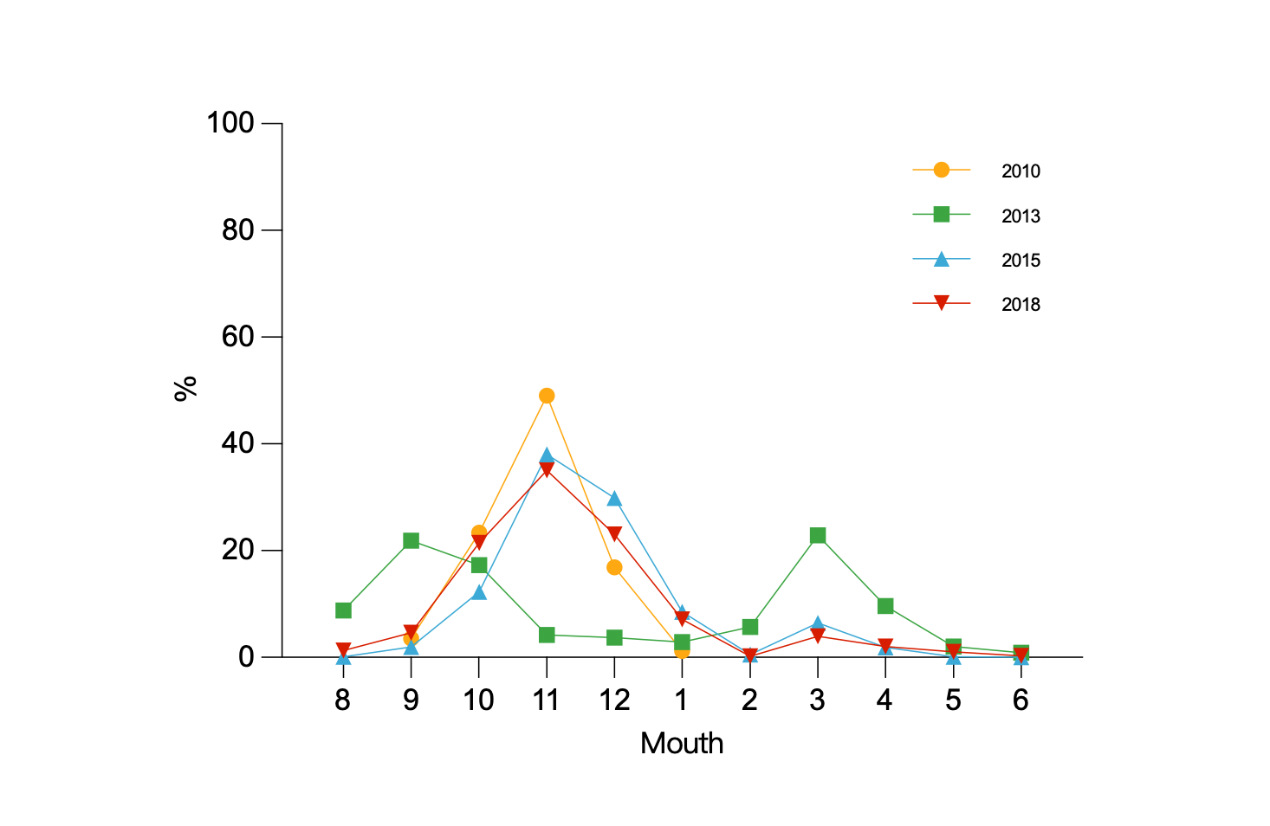


# **Appendix figure 2. Percentages of participants interviewed by month and survey**

# **Appendix table 1. Trends in insufficient physical activity in urban and rural adults in China, 2010-18**

| **Characteristics** | **2010** | **2013** | **2015** | **2018** | ***P* for trend** | **Change in prevalence, 2018 vs. 2010** | ***P* for change** |
| --- | --- | --- | --- | --- | --- | --- | --- |
| **Urban** |  |  |  |  |  |  |  |
| Sex |  |  |  |  |  |  |  |
| Men | 21.7(19.8,23.6) | 20.6(18.8,22.4) | 24.1(22.8,25.3) | 24.3(22.6,26.0) | 0.005 | 2.6(0.2 to 4.9) | <0.001 |
| Women | 15.9(13.9,17.9) | 14.5(13.0,16.0) | 18.7(17.4,20.1) | 19.8(17.7,21.8) | <0.001 | 3.9(1.0 to 6.7) | 0.008 |
| Age group (years) |  |  |  |  |  |  |  |
| 18-34 | 22.0(19.9 to 24.2) | 21.0(18.5 to 23.5) | 25.6(23.9 to 27.2) | 25.6(23.5 to 27.7) | 0.002 | 3.6(0.7 to 6.4) | <0.001 |
| 35-49 | 16.9(14.8 to 19.0) | 15.8(14.3 to 17.3) | 19.4(18.2 to 20.6) | 20.5(18.3 to 22.7) | 0.003 | 3.6(0.6 to 6.6) | <0.001 |
| 50-64 | 13.8(12.0 to 15.7) | 12.9(11.6 to 14.2) | 16.2(14.9 to 17.6) | 18.0(16.2 to 19.9) | <0.001 | 4.2(1.7 to 6.8) | 0.001 |
| ≥65 | 22.8(20.4 to 25.3) | 20.2(18.5 to 22.0) | 22.6(20.3 to 24.9) | 21.3(19.9 to 22.8) | 0.51 | -1.5(-4.2 to 1.2) | 0.28 |
| Ethnicity |  |  |  |  |  |  |  |
| Han | 18.7(16.9 to 20.6) | 17.5(16.0 to 19.1) | 21.4(20.3 to 22.5) | 22.1(20.3 to 23.9) | <0.001 | 3.4(0.90 to 5.8) | 0.008 |
| Others | 20.1(14.4 to 25.8) | 16.8(12.6 to 21.0) | 22.9(19.2 to 26.5) | 21.2(17.3 to 25.1) | 0.48 | 1.1(-5.6 to 7.8) | 0.74 |
| Education |  |  |  |  |  |  |  |
| Secondary school or less | 17.8(15.9 to 19.8) | 15.7(14.3 to 17.0) | 21.0(19.5 to 22.5) | 20.5(19.1 to 21.9) | 0.003 | 2.7(0.2 to 5.1) | 0.031 |
| High school | 18.3(16.3 to 20.3) | 17.6(15.3 to 19.9) | 20.9(19.3 to 22.4) | 22.2(19.2 to 25.3) | 0.02 | 3.9(0.3 to 7.5) | <0.001 |
| College or above | 22.5(19.5 to 25.5) | 22.7(19.9 to 25.4) | 22.9(21.1 to 24.8) | 24.7(21.7 to 27.6) | 0.23 | 2.2(1.7 to 6.0) | 0.27 |
| Occupation |  |  |  |  |  |  |  |
| Agriculture-related work | 13.4(11.0 to 15.8) | 12.1(10.5 to 13.8) | 19.0(16.4 to 21.6) | 18.8(16.5 to 21.1) | <0.001 | 5.4(2.0 to 8.8) | 0.002 |
| Other manual work | 21.8(18.8 to 24.8) | 19.9(16.5 to 23.4) | 21.7(18.1 to 25.3) | 23.6(20.3 to 27.0) | 0.34 | 1.8(-2.8 to 6.4) | 0.43 |
| Non-manual work | 20.8(18.7 to 22.9) | 19.4(17.7 to 21.2) | 22.7(21.4 to 23.9) | 23.7(21.4 to 26.0) | 0.02 | 2.9(0.2 to 5.9) | 0.06 |
| Unemployed/students | 22.8(19.5 to 26.0) | 21.4(18.0 to 24.8) | 25.2(21.7 to 28.6) | 23.5(19.9 to 27.1) | 0.47 | 1.7(-0.7 to 4.1) | 0.76 |
| Retired | 13.5(11.4 to 15.6) | 13.0(10.9 to 15.2) | 14.1(12.8 to 15.4) | 15.2(13.8 to 16.6) | 0.18 | -0.7(-3.9 to 5.3) | 0.17 |
| Income per capita (¥) |  |  |  |  |  |  |  |
| Q1 | 17.6(14.4,20.8) | 14.1(12.1,16.1) | 21.6(19.0,24.3) | 23.1(19.7,26.5) | <0.001 | 5.5(1.2 to 9.7) | 0.012 |
| Q2 | 17.5(15.4,19.6) | 15.4(13.2,17.7) | 22.2(20.2,24.2) | 22.2(19.6,24.7) | <0.001 | 4.7(1.3 to 8.0) | 0.007 |
| Q3 | 17.6(15.6,19.6) | 17.4(15.3,19.5) | 20.2(18.6,21.9) | 19.1(17.5,20.6) | <0.001 | 1.5(-1.0 to 4.0) | 0.25 |
| Q4 | 18.6(16.0,21.1) | 17.3(15.4,19.2) | 20.5(19.0,22.0) | 22.3(20.2,24.3) | <0.001 | 3.7(0.4 to 7.0) | 0.03 |
| Refused/do not konw | 21.4(18.8,24.0) | 20.2(18.1,22.3) | 23.7(21.7,25.7) | 24.1(19.5,28.7) | 0.012 | 2.7(-2.5 to 7.9) | 0.30 |
| BMI category (kg/m^2^) |  |  |  |  |  |  |  |
| <18.5 | 22.2(19.0 to 25.4) | 23.4(19.2 to 27.6) | 27.7(24.2 to 31.2) | 27.5(22.9 to 32.0) | 0.04 | 5.3(-0.4 to 11.0) | 0.07 |
| 18.5-24.9 | 18.0(16.1 to 19.8) | 17.0(15.3 to 18.7) | 21.2(19.9 to 22.5) | 21.4(19.7 to 23.1) | <0.001 | 3.4(1.0 to 5.8) | 0.006 |
| 25.0-29.9 | 19.6(17.5 to 21.6) | 17.1(15.6 to 18.6) | 19.9(18.7 to 21.2) | 22.4(20.0 to 24.8) | 0.02 | 2.8(-0.2 to 5.9) | 0.07 |
| ≥30.0 | 21.0(17.9 to 24.2) | 19.2(16.9 to 21.5) | 21.9(19.7 to 24.1) | 21.5(18.1 to 24.9) | 0.60 | 0.5(-4.2 to 5.2) | 0.83 |
| **Rural** |  |  |  |  |  |  |  |
| Sex |  |  |  |  |  |  |  |
| Men | 18.8(16.6 to 21.0) | 16.1(14.7 to 17.5) | 22.1(20.1 to 24.0) | 24.6(22.5 to 26.6) | <0.001 | 5.8(2.9 to 8.7) | <0.001 |
| Women | 15.5(13.4 to 17.5) | 14.1(12.3 to 15.9) | 19.9(18.0 to 21.8) | 20.7(18.6 to 22.8) | <0.001 | 5.2(2.5 to 7.9)* | <0.001 |
| Age group (years) |  |  |  |  |  |  |  |
| 18-34 | 17.9(15.5 to 20.3) | 16.7(14.7 to 18.8) | 24.9(22.5 to 27.2) | 26.7(23.9 to 29.4) | <0.001 | 8.8(5.2 to 12.4) | <0.001 |
| 35-49 | 13.5(11.6 to 15.3) | 11.6(10.2 to 12.9) | 16.9(15.0 to 18.8) | 19.3(17.2 to 21.3) | <0.001 | 5.8(3.1 to 8.5) | <0.001 |
| 50-64 | 14.1(11.9 to 16.4) | 11.6(10.2 to 13.0) | 16.0(14.2 to 17.7) | 18.4(16.7 to 20.1) | <0.001 | 4.3(1.6 to 6.9) | 0.002 |
| ≥65 | 31.3(28.2 to 34.5) | 26.7(24.4 to 29.1) | 30.7(28.1 to 33.2) | 28.8(26.6 to 30.9) | 0.40 | -2.6(-6.1 to 0.9) | 0.15 |
| Ethnicity |  |  |  |  |  |  |  |
| Han | 17.5(15.5 to 19.6) | 15.0(13.8 to 16.3) | 21.2(19.2 to 23.2) | 23.1(20.9 to 25.2) | <0.001 | 5.5(2.6 to 8.4) | <0.001 |
| Others | 14.2(9.2 to 19.2) | 15.8(10.1 to 21.5) | 19.5(15.9 to 23.0) | 20.0(15.9 to 24.1) | 0.006 | 5.8(2.1 to 9.5) | <0.001 |
| Education (years) |  |  |  |  |  |  |  |
| Secondary school or less | 16.3(14.2 to 18.4) | 14.4(13.0 to 15.8) | 20.3(18.5 to 22.1) | 21.6(19.7 to 23.5) | <0.001 | 5.3(2.7 to 8.0) | <0.001 |
| High school | 20.8(18.3 to 23.4) | 18.4(16.0 to 20.7) | 23.5(20.5 to 26.4) | 25.4(22.3 to 28.5) | 0.009 | 4.6(0.4 to 8.7) | 0.030 |
| College or above | 27.5(22.3 to 32.7) | 24.3(20.2 to 28.5) | 28.7(23.4 to 34.0) | 29.6(25.3 to 33.9) | 0.49 | 2.1(-4.7 to 8.8) | 0.54 |
| Occupation |  |  |  |  |  |  |  |
| Agriculture-related work | 13.7(11.7 to 15.6) | 12.6(10.9 to 14.3) | 17.8(15.4 to 20.1) | 19.4(17.1 to 21.6) | <0.001 | 5.7(2.9 to 8.5) | <0.001 |
| Other manual work | 17.9(14.1 to 21.7) | 17.7(14.5 to 20.8) | 22.6(18.5 to 26.7) | 24.5(20.3 to 28.7) | 0.002 | 6.5(1.6 to 11.5) | 0.009 |
| Non-manual work | 23.0(20.0 to 26.0) | 18.1(16.3 to 19.9) | 24.2(22.1 to 26.3) | 25.3(23.2 to 27.4) | 0.03 | 2.3(-1.3 to 5.8) | 0.21 |
| Unemployed/students | 31.1(22.8 to 39.4) | 26.8(21.8 to 31.9) | 32.1(28.7 to 35.5) | 32.0(27.6 to 36.4) | 0.88 | 0.9(-8.2 to 10.0) | 0.85 |
| Retired | 28.5(23.5 to 33.4) | 23.7(17.4 to 30.0) | 27.8(21.7 to 34.0) | 26.1(21.9 to 30.3) | 0.61 | -2.4(-9.0 to 4.3) | 0.48 |
| Income per capita (¥) |  |  |  |  |  |  |  |
| Q1 | 17.0(13.8 to 20.1) | 15.0(13.1 to 16.9) | 21.8(19.5 to 24.1) | 23.1(20.7 to 25.6) | <0.001 | 6.2(2.7 to 9.7) | <0.001 |
| Q2 | 15.9(13.1 to 18.6) | 13.3(11.9 to 14.7) | 20.0(17.7 to 22.2) | 22.0(18.4 to 25.5) | <0.001 | 6.1(1.6 to 10.6) | <0.001 |
| Q3 | 15.2(13.1 to 17.4) | 14.4(13.0 to 15.8) | 20.3(17.9 to 22.6) | 20.6(18.3 to 22.9) | <0.001 | 5.4(2.5 to 8.3) | <0.001 |
| Q4 | 18.6(15.7 to 21.4) | 16.3(14.4 to 18.2) | 19.3(17.3 to 21.3) | 23.7(20.8 to 26.6) | 0.008 | 5.1(1.1 to 9.1) | 0.014 |
| Refused/do not konw | 19.0(16.0 to 21.9) | 17.1(13.1 to 21.1) | 23.1(20.3 to 25.9) | 23.7(21.1 to 26.4) | 0.005 | 4.8(0.7 to 8.9) | 0.02 |
| BMI category (kg/m^2^) |  |  |  |  |  |  |  |
| <18.5 | 20.6(17.5 to 23.6) | 20.3(16.9 to 23.6) | 24.7(21.9 to 27.5) | 26.6(22.0 to 31.3) | 0.01 | 6.1(0.6 to 11.5) | 0.031 |
| 18.5-24.9 | 16.4(14.3 to 18.5) | 14.4(13.0 to 15.7) | 20.1(18.3 to 22.0) | 21.9(20.0 to 23.8) | <0.001 | 5.5(2.9 to 8.1) | <0.001 |
| 25.0-29.9 | 17.8(15.7 to 19.9) | 15.1(13.4 to 16.8) | 20.7(18.5 to 22.9) | 22.3(20.0 to 24.5) | <0.001 | 4.4(1.5 to 7.3) | 0.003 |
| ≥30.0 | 19.1(16.7 to 21.5) | 19.2(16.2 to 22.2) | 21.5(18.8 to 24.2) | 25.2(22.2 to 28.2) | 0.001 | 6.1(2.3 to 9.9) | 0.002 |

Values are mean (95%CI). All calculations are weighted, accounting for the multistage cluster sampling design. CI=confidence interval. MVPA= moderate to vigorous insufficient physical activity.

# **Appendix table 2. Trends in adults undertaking 150-299 min/week of MVPA in China, 2010 -18**

| **Characteristics** | **2010** | **2013** | **2015** | **2018** | ***P* for trend** | **Change in prevalence to 2018 vs. 2010** | ***P* for change** |
| --- | --- | --- | --- | --- | --- | --- | --- |
| **Total** | 10.6(9.9 to 11.4) | 9.5(8.9 to 10.1) | 10.8(10.2 to 11.4) | 10.9(10.3 to 11.5) | 0.23 | 0.3(-0.7 to 1.2) | 0.57 |
| Sex |  |  |  |  |  |  |  |
| Men | 10.6(9.8 to 11.4) | 9.3(8.6 to 9.9) | 10.2(9.5 to 10.9) | 10.2(9.5 to 10.9) | 0.78 | -0.4(-1.4 to 0.7) | 0.48 |
| Women | 10.7(9.9 to 11.5) | 9.7(8.9 to 10.5) | 11.4(10.7 to 12.2) | 11.6(10.9 to 12.3) | 0.02 | 0.9(-0.1 to 2.0) | 0.09 |
| Age group (years) |  |  |  |  |  |  |  |
| 18-34 | 13.0(11.9 to 14.1) | 11.0(10.1 to 11.9) | 13.4(12.2 to 14.7) | 13.7(12.5 to 14.9) | 0.14 | 0.7(-1.0 to 2.3) | 0.43 |
| 35-49 | 9.1(8.3 to 10.0) | 8.3(7.5 to 9.2) | 9.8(9.1 to 10.5) | 9.8(9.2 to 10.5) | 0.044 | 0.7(-0.3 to 1.7) | 0.16 |
| 50-64 | 8.5(7.8 to 9.2) | 7.8(7.3 to 8.4) | 8.2(7.7 to 8.7) | 8.1(7.6 to 8.7) | 0.56 | -0.3(-1.1 to 0.5) | 0.41 |
| ≥65 | 11.8(10.7 to 12.9) | 11.3(10.2 to 12.5) | 10.5(9.9 to 11.1) | 10.5(9.9 to 11.1) | 0.022 | -1.3(-2.5 to -0.0) | 0.05 |
| Geographic location |  |  |  |  |  |  |  |
| Urban | 13.0(12.1 to 13.9) | 11.7(10.8 to 12.6) | 12.7(11.9 to 13.6) | 13.1(12.2 to 13.9) | 0.66 | 0.1(-1.2 to 1.3) | 0.94 |
| Rural | 8.6(7.8 to 9.4) | 7.6(7.0 to 8.2) | 8.7(8.2 to 9.3) | 8.6(8.0 to 9.2) | 0.50 | 0.0(-0.9 to 0.9) | 0.97 |
| Ethnicity |  |  |  |  |  |  |  |
| Han | 10.9(10.1 to 11.7) | 9.8(9.1 to 10.4) | 11.0(10.3 to 11.7) | 11.1(10.4 to 11.7) | 0.38 | 0.1(-0.8 to 1.1) | 0.77 |
| Others | 7.7(6.3 to 9.1) | 6.7(5.5 to 7.9) | 8.3(7.3 to 9.4) | 9.4(8.2 to 10.5) | 0.016 | 1.7(0.1 to 3.2) | 0.036 |
| Education (years) |  |  |  |  |  |  |  |
| Secondary school or less | 9.0(8.2 to 9.7) | 8.0(7.5 to 8.6) | 8.9(8.3 to 9.5) | 8.7(8.1 to 9.2) | 0.81 | -0.3(-1.2 to 0.6) | 0.49 |
| High school | 13.2(12.0 to 14.5) | 11.3(10.4 to 12.2) | 12.6(11.4 to 13.7) | 12.6(11.5 to 13.7) | 0.67 | -0.7(-2.3 to 0.9) | 0.42 |
| College or above | 17.7(16.4 to 19.1) | 16.5(15.0 to 18.0) | 17.6(16.2 to 19.0) | 17.4(16.1 to 18.8) | 0.97 | -0.3(-2.1 to 1.5) | 0.74 |
| Occupation |  |  |  |  |  |  |  |
| Agriculture-related work | 7.2(6.4 to 8.1) | 6.5(6.0 to 7.1) | 7.3(6.8 to 7.8) | 7.1(6.3 to 8.0) | 0.94 | -0.1(-1.2 to 1.0) | 0.85 |
| Other manual work | 12.3(9.6 to 15.0) | 9.9(8.5 to 11.3) | 8.7(7.6 to 9.8) | 8.7(7.5 to 10.0) | 0.010 | -3.6(-6.5 to 0.6) | 0.02 |
| Non-manual work | 13.6(12.6 to 14.7) | 11.7(10.7 to 12.7) | 13.3(12.3 to 14.3) | 13.2(12.4 to 14.0) | 0.99 | -0.5(-1.8 to 0.8) | 0.46 |
| Unemployed/students | 14.4(13.0 to 15.8) | 14.3(12.4 to 16.3) | 14.2(12.7 to 15.7) | 14.1(12.0 to 16.2) | 0.81 | -0.3(-2.9 to 2.3) | 0.83 |
| Retired | 10.5(9.4 to 11.6) | 10.3(9.3 to 11.3) | 10.5(9.5 to 11.6) | 11.5(10.5 to 12.5) | 0.24 | 1.0(-0.6 to 2.6) | 0.23 |
| Income per capita (¥) |  |  |  |  |  |  |  |
| Q1 | 8.6(7.5 to 9.7) | 7.9(7.0 to 8.9) | 9.7(8.7 to 10.7) | 9.3(8.3 to 10.3) | 0.10 | 0.7(-0.6 to 2.0) | 0.30 |
| Q2 | 8.7(7.8 to 9.6) | 8.4(7.6 to 9.3) | 9.3(8.4 to 10.2) | 9.9(8.7 to 11.2) | 0.08 | 1.3(-0.4 to 2.9) | 0.13 |
| Q3 | 10.7(9.8 to 11.6) | 9.4(8.7 to 10.2) | 11.1(9.9 to 12.3) | 10.2(9.3 to 11.1) | 0.91 | -0.5(-1.8 to 0.7) | 0.40 |
| Q4 | 12.6(11.5 to 13.7) | 11.5(10.0 to 12.9) | 12.1(11.2 to 12.9) | 13.1(12.2 to 14.0) | 0.35 | 0.5(-0.9 to 1.8) | 0.49 |
| Refused/do not konw | 11.8(10.5 to 13.1) | 10.1(9.1 to 11.1) | 11.1(10.1 to 12.0) | 10.9(9.9 to 12.0) | 0.45 | -0.8(-2.5 to 0.8) | 0.31 |
| BMI category (kg/m^2^) |  |  |  |  |  |  |  |
| <18.5 | 12.4(11.0 to 13.9) | 10.4(8.6 to 12.2) | 11.2(9.7 to 12.7) | 14.5(11.3 to 17.7) | 0.22 | 2.1(-1.4 to 5.6) | 0.24 |
| 18.5-24.9 | 10.6(9.8 to 11.4) | 9.5(8.8 to 10.2) | 10.9(10.1 to 11.7) | 10.8(10.1 to 11.6) | 0.38 | 0.2(-0.9 to 1.3) | 0.69 |
| 25.0-29.9 | 10.2(9.4 to 11.0) | 9.4(8.6 to 10.2) | 10.4(9.7 to 11.0) | 10.7(9.8 to 11.5) | 0.29 | 0.4(-0.8 to 1.6) | 0.50 |
| ≥30.0 | 11.3(10.0 to 12.6) | 9.0(8.0 to 9.9) | 11.6(9.9 to 13.3) | 10.6(9.3 to 11.9) | 0.95 | -0.7(-2.4 to 1.0) | 0.40 |

Values are % (95% CI). All calculations are weighted, accounting for the multistage cluster sampling design. CI=confidence interval. MVPA= moderate to vigorous insufficient physical activity.

# **Appendix table 3. Trends in percentages of adults participating in domain-specific MVPA in China, 2010-18**

| **Characteristics** | **2010** | **2013** | **2015** | **2018** | ***P* for trend** | **Change in prevalence to 2018 vs. 2010** | ***P* for change** |
| --- | --- | --- | --- | --- | --- | --- | --- |
| **Work-related** |  |  |  |  |  |  |  |
| Total | 79.6(77.8 to 81.5) | 79.2(77.7 to 80.7) | 71.8(70.2 to 73.3) | 66.8(64.9 to 68.7) | <0.001 | -12.8(-15.3 to -10.3) | <0.001 |
| Gender |  |  |  |  |  |  |  |
| Men | 75.3(73.3 to 77.3) | 75.5(73.8 to 77.2) | 67.6(66.0 to 69.3) | 63.1(61.1 to 65.0) | <0.001 | -12.2(-14.8 to -9.6) | <0.001 |
| Women | 84.1(82.2 to 85.9) | 83.0(81.5 to 84.6) | 76.0(74.4 to 77.6) | 70.6(68.6 to 72.7) | <0.001 | -13.4(-16.1 to -10.8) | <0.001 |
| Age group (years) |  |  |  |  |  |  |  |
| 18-34 | 78.2(75.9 to 80.4) | 76.4(74.2 to 78.5) | 67.1(65.1 to 69.0) | 61.1(58.7 to 63.5) | <0.001 | -17.1(-20.4 to -13.8) | <0.001 |
| 35-49 | 83.5(81.7 to 85.3) | 83.8(82.4 to85.2) | 77.1(75.5 to 78.7) | 71.8(69.7 to 73.9) | <0.001 | -11.7(-14.2 to -9.1) | <0.001 |
| 50-64 | 82.2(80.3 to 84.2) | 82.7(81.2 to 84.1) | 77.2(75.6 to 78.7) | 72.0(70.1 to 73.9) | <0.001 | -10.2(-12.7 to -7.8) | <0.001 |
| ≥65 | 68.0(65.7 to 70.3) | 68.0(66.0 to 70.1) | 60.7(58.6 to 62.8) | 60.2(58.3 to 62.2) | <0.001 | -7.7(-10.6 to -4.9) | <0.001 |
| Geographic location |  |  |  |  |  |  |  |
| Urban | 75.7(73.5 to 77.9) | 74.4(72.3 to 76.5) | 68.2(66.3 to 70.0) | 63.2(61.0 to 65.5) | <0.001 | -12.5(-15.5 to -9.4) | <0.001 |
| Rural | 83.0(80.9 to 85.2) | 83.3(81.8 to 84.8) | 75.6(73.6 to 77.6) | 70.7(68.2 to 73.1) | <0.001 | -12.4(-15.5 to -9.3) | <0.001 |
| **Transport-related** |  |  |  |  |  |  |  |
| Total | 57.0(54.3 to 59.7) | 52.6(50.3 to 55.0) | 53.9(51.7 to 56.1) | 52.2(50.1 to 54.2) | 0.014 | -4.8(-8.2 to -1.4) | 0.005 |
| Gencer |  |  |  |  |  |  |  |
| Men | 53.1(50.2 to 56.1) | 48.7(46.3 to 51.0) | 49.6(47.3 to 51.8) | 49.2(47.1 to 51.4) | 0.06 | -3.9(-7.5 to -0.2) | 0.040 |
| Women | 60.8(58.2 to 63.4) | 56.5(54.0 to 59.0) | 58.1(55.8 to 60.4) | 55.0(52.7 to 57.2) | 0.004 | -5.8(-9.2 to -2.4) | <0.001 |
| Age group (years) |  |  |  |  |  |  |  |
| 18-34 | 55.6(52.5 to 58.8) | 50.6(47.6 to 53.7) | 51.5(48.6 to 54.5) | 51.6(49.0 to 54.1) | 0.07 | -4.1(-8.0 to -0.1) | 0.045 |
| 35-49 | 56.6(53.7 to 59.6) | 51.7(49.2 to 54.1) | 53.7(51.3 to 56.1) | 52.6(50.2 to 54.9) | 0.08 | -4.1(-7.9 to -0.3) | 0.037 |
| 50-64 | 62.0(59.0 to 65.0) | 56.7(54.4 to 59.1) | 57.8(55.7 to 59.9) | 52.4(50.3 to 54.6) | <0.001 | -9.5(-13.2 to -5.9) | <0.001 |
| ≥65 | 52.8(49.6 to 56.0) | 54.0(51.5 to 56.5) | 54.1(51.8 to 56.4) | 52.3(49.9 to 54.7) | 0.83 | -0.5(-4.3 to 3.3) | 0.79 |
| Geographic location |  |  |  |  |  |  |  |
| Urban | 60.9(58.0 to 63.9) | 57.0(54.4 to 59.6) | 59.1(56.5 to 61.6) | 56.8(53.9 to 59.7) | 0.08 | -4.1(-8.0 to -0.3) | 0.036 |
| Rural | 53.8(50.5 to 57.1) | 49.2(46.4 to 51.9) | 48.9(46.2 to 51.5) | 47.8(45.5 to 50.0) | 0.007 | -6.1(-10.2 to -1.9) | 0.005 |
| **Recreation-related** |  |  |  |  |  |  |  |
| Total | 14.2(12.5 to 15.9) | 17.0(15.3 to 18.7) | 15.5(13.9 to 17.2) | 17.2(16.0 to 18.4) | 0.014 | 3.0(1.0 to 4.9) | 0.003 |
| Gender |  |  |  |  |  |  |  |
| Men | 15.7(13.9 to17.4) | 17.7(15.8 to 19.5) | 16.5(14.5 to 18.4) | 18.6(17.0 to 20.2) | 0.029 | 2.9(0.7 to 5.1) | 0.010 |
| Women | 12.7(11.0 to14.5) | 16.3(14.6 to 17.9) | 14.6(13.2 to 16.1) | 15.8(14.7 to 16.8) | 0.016 | 3.1(1.1 to 5.1) | 0.003 |
| Age group (years) |  |  |  |  |  |  |  |
| 18-34 | 17.7(15.5 to 20.0) | 20.3(17.9 to 22.6) | 18.2(15.9 to 20.6) | 20.1(18.0 to 22.2) | 0.23 | 2.4(0.6 to 5.3) | 0.11 |
| 35-49 | 12.8(11.0 to 14.5) | 16.3(14.6 to 18.0) | 16.1(14.2 to 17.9) | 17.9(16.7 to 19.2) | <0.001 | 5.2(3.2 to 7.2) | <0.001 |
| 50-64 | 12.8(10.8 to 14.8) | 15.6(14.0 to 17.3) | 13.8(12.7 to 14.9) | 14.8(13.9 to 15.7) | 0.17 | 2.0(0.04 to 4.2) | 0.05 |
| ≥65 | 10.4(8.5 to 12.3) | 11.6(10.1 to 13.0) | 9.3(8.3 to 10.2) | 10.6(9.7 to 11.4) | 0.71 | 0.2(-1.9 to 2.2) | 0.87 |
| Geographic location |  |  |  |  |  |  |  |
| Urban | 21.4(19.0 to 23.8) | 24.0(21.8 to 26.2) | 21.0(18.6 to 23.4) | 21.2(19.5 to 23.0) | 0.54 | 0.2(-3.0 to 2.7) | 0.91 |
| Rural | 8.2(7.0 to 9.3) | 11.3(9.9 to 12.6) | 10.0(8.9 to 11.0) | 13.0(12.0 to 14.0) | <0.001 | 4.8(3.4 to 6.3)* | <0.001 |

Values are % (95% CI). All calculations are weighted, accounting for the multistage cluster sampling design. CI=confidence interval. MVPA= moderate to vigorous insufficient physical activity.

# **Appendix table 4. Trends in mean min/week of domain-specific MVPA among adults in China, 2010-18**

| **Characteristics** | **2010** | **2013** | **2015** | **2018** | ***P* for trend** |
| --- | --- | --- | --- | --- | --- |
| **Total** |  |  |  |  |  |
| Total | 1274.2 (1563.4) | 1417.6 (1715.0) | 1364.2 (1831.7) | 1281.5 (1787.6) | 0.84 |
| Gender |  |  |  |  |  |
| Men | 1380.5 (1717.3) | 1620.1 (1973.5) | 1532.7 (2042.8) | 1435.4 (2004.8) | 0.68 |
| Women | 1165.9 (1380.8) | 1211.0 (1372.5) | 1192.3 (1569.0) | 1125.7 (1521.2) | 0.35 |
| Age group (years) |  |  |  |  |  |
| 18-34 | 1101.4 (1452.6) | 1237.8 (1566.1) | 1086.7 (1606.7) | 1004.7 (1519.0) | 0.008 |
| 35-49 | 1499.3 (1709.3) | 1669.6 (1921.3) | 1599.8 (2011.2) | 1483.7 (1986.8) | 0.57 |
| 50-64 | 1436.8 (1709.3) | 1580.0 (1757.0) | 1664.5 (1996.7) | 1573.1 (1964.9) | 0.007 |
| ≥65 | 845.4 (1147.8) | 930.9 (1183.9) | 973.7 (1352.5) | 1003.3 (1381.8) | <0.001 |
| Geographic location |  |  |  |  |  |
| Urban | 983.9 (1253.2) | 1105.5 (1408.6) | 1113.3 (1579.8) | 1023.5 (1481.7) | 0.39 |
| Rural | 1526.2 (1751.0) | 1683.1 (1897.8) | 1633.5 (2034.1) | 1557.7 (2029.6) | 0.79 |
| **Work-related** |  |  |  |  |  |
| Total | 1020.8 (1482.3) | 1151.8 (1649.7) | 1074.8 (1748.9) | 958.4 (1673.1) | 0.09 |
| Gender |  |  |  |  |  |
| Men | 1117.3 (1653.6) | 1346.5 (1917.9) | 1230.3 (1978.8) | 1098.1 (1905) | 0.34 |
| Women | 922.5 (1276.7) | 953.1 (1291) | 916.1 (1460.9) | 817.1 (1385.6) | 0.014 |
| Age group (years) |  |  |  |  |  |
| 18-34 | 858.0 (1353.5) | 977.0 (1505.9) | 806.9 (1520.1) | 672.1 (1382.5) | <0.001 |
| 35-49 | 1259.9 (1643.4) | 1424.1 (1856.1) | 1329 (1939.7) | 1177.5 (1876.5) | 0.09 |
| 50-64 | 1150.1 (1531.8) | 1282.8 (1694.4) | 1342.6 (1908) | 1240.5 (1864.6) | 0.07 |
| ≥65 | 582.0 (1046.4) | 647.9 (1058.5) | 660 (1228.6) | 678.0 (1244.4) | 0.01 |
| Geographic location |  |  |  |  |  |
| Urban | 686.2 (1179.4) | 788.5 (1324.2) | 780.7 (1490.5) | 673.0 (1365.7) | 0.69 |
| Rural | 1311.3 (1647.8) | 1460.7 (1826.7) | 1390.5 (1940.3) | 1264 (1902.4) | 0.40 |
| **Transport-related** |  |  |  |  |  |
| Total | 190.5 (311) | 185.2 (315.8) | 212.8 (387.5) | 234.4 (407.6) | <0.001 |
| Gender |  |  |  |  |  |
| Men | 188.9 (317.1) | 180.3 (322) | 213.8 (404.5) | 233.4 (416.6) | <0.001 |
| Women | 192.0 (304.6) | 190.2 (309.2) | 211.8 (369.4) | 235.4 (398.4) | <0.001 |
| Age group (years) |  |  |  |  |  |
| 18-34 | 169.9 (295.1) | 165.4 (291.6) | 191.3 (371.3) | 222.1 (386.1) | <0.001 |
| 35-49 | 185.9 (308.6) | 173.2 (310.6) | 197.4 (371.5) | 223.6 (406.7) | <0.001 |
| 50-64 | 222.1 (327.7) | 216.6 (340.7) | 248.7 (421.3) | 256.6 (440.1) | <0.001 |
| ≥65 | 208.0 (327.4) | 222.5 (344.9) | 257.9 (408.4) | 263.4 (409.5) | <0.001 |
| Geographic location |  |  |  |  |  |
| Urban | 207.5 (307.5) | 206.8 (317.68) | 229.8 (381) | 243.2 (377.5) | <0.001 |
| Rural | 175.7 (313.3) | 166.9 (313.1) | 194.5 (393.6) | 225.1 (437.4) | <0.001 |
| **Recreation-related** |  |  |  |  |  |
| Total | 62.9 (253.3) | 80.7 (281.9) | 76.6 (273.4) | 88.6 (307.3) | <0.001 |
| Gender |  |  |  |  |  |
| Men | 74.3 (267.2) | 93.3 (322.5) | 88.5 (300.1) | 103.9 (337.6) | <0.001 |
| Women | 51.4 (237.9) | 67.7 (232.6) | 64.4 (242.5) | 73.1 (272.5) | <0.001 |
| Age group (years) |  |  |  |  |  |
| 18-34 | 73.6 (284.2) | 95.4 (287.6) | 88.4 (278.9) | 110.5 (328.9) | <0.001 |
| 35-49 | 53.4 (225.9) | 72.3 (287.5) | 73.4 (275.9) | 82.7 (307.2) | <0.001 |
| 50-64 | 64.5 (264.6) | 80.6 (292.4) | 73.2 (283.1) | 75.9 (294.4) | 0.15 |
| ≥65 | 55.5 (201.6) | 60.5 (218.8) | 55.8 (224.5) | 62.0 (253.6) | 0.42 |
| Geographic location |  |  |  |  |  |
| Urban | 90.1 (255.4) | 110.2 (293) | 102.8 (288.1) | 107.3 (308.6) | 0.05 |
| Rural | 39.3 (249.1) | 55.5 (269.6) | 48.5 (253.7) | 68.6 (304.8) | <0.001 |

Values are mean minutes per week (standard deviation). All calculations are weighted, accounting for the multistage cluster sampling design. CI=confidence interval. MVPA= moderate to vigorous insufficient physical activity.

# **Appendix table 5. Mean domain-specific relative contribution to total MVPA among adults in China, 2010-18**

| **Characteristics** | **2010** | **2013** | **2015** | **2018** | ***P* for trend** |
| --- | --- | --- | --- | --- | --- |
| **Total** |  |  |  |  |  |
| Work-related | 66.5(64.1 to 68.9) | 67.2(65.2 to 69.2) | 60.8(58.9 to 62.7) | 55.2(53.2 to 57.1) | <0.001 |
| Transport-related | 25.9(24.2 to 27.6) | 24.1(22.7 to 25.5) | 31.1(29.7 to 32.6) | 34.8(33.1 to 36.4) | <0.001 |
| Recreation- related | 7.6(6.6 to 8.7) | 8.7(7.8 to 9.6) | 8.1(7.3 to 8.8) | 10.1(9.3 to 10.8) | <0.001 |
| **Men** |  |  |  |  |  |
| Work-related | 63.4(60.7 to 66.2) | 65.3(63 to 67.5) | 58.7(56.6 to 60.8) | 52.6(50.5 to 54.8) | <0.001 |
| Transport-related | 26.9(25 to 28.8) | 24.1(22.7 to 25.6) | 31.6(30.1 to 33.1) | 35.2(33.4 to 36.9) | <0.001 |
| Recreation-related | 9.7(8.4 to 11.0) | 10.6(9.4 to 11.8) | 9.7(8.8 to 10.6) | 12.2(11.2 to 13.2) | 0.008 |
| **Women** |  |  |  |  |  |
| Work-related | 69.5(67.3 to 71.7) | 69.1(67.2 to 71.0) | 62.9(61 to 64.7) | 57.6(55.7 to 59.6) | <0.001 |
| Transport-related | 24.9(23.2 to 26.5) | 24.1(22.6 to 25.6) | 30.6(29.1 to 32.1) | 34.4(32.6 to 36.1) | <0.001 |
| Recreation-related | 5.6(4.8 to 6.5) | 6.8(6.1 to 7.5) | 6.5(5.8 to 7.2) | 8.0(7.4 to 8.6) | <0.001 |
| **Urban** |  |  |  |  |  |
| Work-related | 55.2(52.2 to 58.2) | 55.8(53.4 to 58.1) | 50.8(48.7 to 52.9) | 46(43.5 to 48.5) | <0.001 |
| Transport-related | 32.8(30.7 to 34.8) | 31.1(29.2 to 32.9) | 37.8(36.1 to 39.4) | 41.0(38.8 to 43.2) | <0.001 |
| Recreation-related | 12.1(10.7 to 13.4) | 13.2(11.9 to 14.4) | 11.4(10.4 to 12.4) | 13.0(12.0 to 14.0) | 0.68 |
| **Rural** |  |  |  |  |  |
| Work-related | 76.4(74.6 to 78.1) | 76.8(75.3 to 78.3) | 71.8(70.4 to 73.1) | 65.2(63.4 to 67.0) | <0.001 |
| Transport-related | 19.9(18.3 to 21.4) | 18.3(17.0 to 19.5) | 23.8(22.5 to 25.2) | 27.9(26.3 to 29.5) | <0.001 |
| Recreation-related | 3.8(3.1 to 4.5) | 4.9(4.4 to 5.5) | 4.4(4 to 4.8) | 6.9(6.2 to 7.6) | <0.001 |

Values are % (95% CI). All calculations are weighted, accounting for the multistage cluster sampling design.

# **Appendix table 6. Trends in** **percentages of adults without intensity-specific MVPA in China, 2010-18**

| **Characteristics** | **2010** | **2013** | **2015** | **2018** | ***P* for trend** | **Change in prevalence, 2018 vs. 2010** | ***P* for change** |
| --- | --- | --- | --- | --- | --- | --- | --- |
| **% of no vigorous PA** |  |  |  |  |  |  |  |
| Total | 67.5(65.3 to 69.7) | 70.4(68.8 to 72.1) | 71.6(70.3 to 72.9) | 73.1(71.9 to 74.4) | <0.001 | 5.7(3.3. to 8.0) | <0.001 |
| Gender |  |  |  |  |  |  |  |
| Men | 58.3(55.9 to 60.6) | 59.5(57.6 to 61.4) | 61.8(60.3 to 63.4) | 63.5(61.8 to 65.1) | <0.001 | 5.2(2.6 to 7.9) | <0.001 |
| Women | 76.9(74.6 to 79.2) | 81.5(80.0 to 83.1) | 81.6(80.3 to 82.8) | 82.9(81.8 to 84.1) | <0.001 | 6.1(3.7 to 8.4) | <0.001 |
| Age group (years) |  |  |  |  |  |  |  |
| 18-34 | 67.3(64.9 to 69.7) | 70.2(68.2 to 72.2) | 72.9(71.3 to 74.5) | 72.8(70.7 to 74.8) | <0.001 | 5.5(2.4 to 8.5) | <0.001 |
| 35-49 | 61.7(59.0 to 64.4) | 65.2(63.1 to 67.3) | 66.4(64.7 to 68.1) | 69.6(68.0 to 71.1) | <0.001 | 7.9(5.0 to 10.7) | <0.001 |
| 50-64 | 66.6(63.8 to 69.4) | 70.4(68.4 to 72.5) | 69.7(67.9 to 71.5) | 72.9(71.3 to 74.5) | <0.001 | 6.2(3.3 to 9.2) | <0.001 |
| ≥65 | 86.4(84.5 to 88.2) | 86.4(84.9 to 87.9) | 86.2(85.0 to 87.4) | 85.3(83.8 to 86.8) | 0.32 | 1.1(-3.2 to 1.1) | 0.32 |
| Geographic location |  |  |  |  |  |  |  |
| Urban | 76.4(74.8 to 78.1) | 77.3(75.4 to 79.1) | 75.6(73.7 to 77.5) | 76.5(74.9 to 78.1) | 0.77 | 0.1(-2.0 to 2.2) | 0.93 |
| Rural | 59.7(57.1 to 62.3) | 64.6(62.5 to 66.7) | 67.3(65.4 to 69.1) | 69.6(68.0 to 71.2) | <0.001 | 9.8(6.8 to 12.8) | <0.001 |
| **% of no moderate PA** |  |  |  |  |  |  |  |
| Total | 12.6(11.2 to 14.0) | 12.9(11.8 to 14.0) | 15.2(14.0 to 16.5) | 15.9(14.5 to 17.3) | <0.001 | 3.3(1.2 to 5.3) | 0.002 |
| Gender |  |  |  |  |  |  |  |
| Men | 16.6(14.7 to 18.4) | 17.1(15.7 to 18.4) | 19.3(17.7 to 20.8) | 19.0(17.6 to 20.4) | 0.02 | 2.4(0.1 to 4.8) | 0.044 |
| Women | 8.6(7.4 to 9.8) | 8.6(7.4 to 9.7) | 11.1(10.0 to 12.2) | 12.7(11.2 to 14.3) | <0.001 | 4.1(2.1 to 6.1) | <0.001 |
| Age group (years) |  |  |  |  |  |  |  |
| 18-34 | 13.4(11.7 to 15.1) | 14.3(12.7 to 15.9) | 17.1(15.4 to 18.7) | 16.9(15.2 to 18.6) | 0.003 | 3.5(1.0 to 6.1) | 0.007 |
| 35-49 | 11.7(10.3 to 13.1) | 11.7(10.7 to 12.8) | 13.8(12.5 to 15.0) | 14.6(13.0 to 16.2) | 0.003 | 2.9(0.8 to 5.0) | 0.008 |
| 50-64 | 10.3(8.8 to 11.8) | 10.5(9.4 to 11.5) | 12.5(11.4 to 13.5) | 15.0(13.6 to 16.4) | <0.0001 | 4.7(2.7 to 6.7) | <0.001 |
| ≥65 | 17.1(15.0 to 19.3) | 16.1(14.5 to 17.6) | 18.9(17.3 to 20.6) | 18.1(16.7 to 19.4) | 0.22 | 0.9(-1.5 to 3.4) | 0.46 |
| Geographic location |  |  |  |  |  |  |  |
| Urban | 11.5(10.0 to 12.9) | 12.5(11.2 to 13.8) | 13.3(12.1 to 14.5) | 14.1(12.3 to 15.9) | 0.02 | 2.6(0.3 to 4.9) | 0.025 |
| Rural | 13.6(11.7 to 15.5) | 13.2(11.7 to 14.6) | 17.3(15.5 to 19.1) | 17.8(16.3 to 19.3) | <0.001 | 4.2(1.7 to 6.6) | <0.001 |
| **% of no MVPA** |  |  |  |  |  |  |  |
| Total | 48.3(43.1 to 53.5) | 45.5(40.9 to 50.0) | 51.3(46.6 to 55.9) | 52.7(48.8 to 56.5) | 0.07 | 4.4(-1.7 to 10.5) | 0.16 |
| Gender |  |  |  |  |  |  |  |
| Men | 50.4(45.2 to 55.5) | 48.5(44.0 to 53.1) | 53.6(48.8 to 58.4) | 52.9(49.2 to 56.7) | 0.22 | 2.5(-3.3 to 8.4) | 0.39 |
| Women | 45.6(39.8 to 51.3) | 41.9(36.9 to 47.0) | 48.5(43.8 to 53.3) | 52.4(48.0 to 56.8) | 0.02 | 6.8(-0.2 to 13.8) | 0.06 |
| Age group (years) |  |  |  |  |  |  |  |
| 18-34 | 43.3(37.4 to 49.1) | 44.0(38.1 to 49.8) | 50.8(45.0 to 56.7) | 51.0(46.4 to 55.6) | 0.01 | 7.7(0.6 to 14.8) | 0.033 |
| 35-49 | 47.9(42.2 to 53.7) | 42.3(37.8 to 46.7) | 45.6(40.6 to 50.6) | 48.0(43.4 to 52.7) | 0.71 | 0.1(-7.1 to 7.4) | 0.97 |
| 50-64 | 44.7(38.3 to 51.2) | 40.4(35.8 to 45.0) | 47.6(43.7 to 51.5) | 53.9(50.0 to 57.9) | 0.004 | 9.2(2.0 to 16.4) | 0.013 |
| ≥65 | 68.3(62.6 to 74.0) | 64.6(60.0 to 69.1) | 71.9(68.3 to 75.5) | 69.2(66.1 to 72.4) | 0.41 | 0.9(-5.4 to 7.3) | 0.77 |
| Geographic location |  |  |  |  |  |  |  |
| Urban | 37.2(31.8 to 42.5) | 37.6(32.9 to 42.2) | 40.6(35.6 to 45.6) | 43.9(38.8 to 48.9) | 0.04 | 6.7(-0.3 to 13.7) | 0.06 |
| Rural | 63.5(57.5 to 69.5) | 56.2(50.5 to 62.0) | 67.0(62.7 to 71.4) | 64.0(60.5 to 67.6) | 0.36 | 0.5(-6.3 to 7.3) | 0.88 |

Values are % (95% CI). All calculations included are weighted, accounting for the multistage cluster sampling design. CI=confidence interval. MVPA= moderate or vigorous insufficient physical activity.
